# Strategies to Improve the Impact of Artificial Intelligence on Health Equity: Scoping Review

**DOI:** 10.2196/42936

**Published:** 2023-02-07

**Authors:** Carl Thomas Berdahl, Lawrence Baker, Sean Mann, Osonde Osoba, Federico Girosi

**Affiliations:** 1 RAND Corporation Santa Monica, CA United States; 2 Department of Medicine Cedars-Sinai Medical Center Los Angeles, CA United States; 3 Department of Emergency Medicine Cedars-Sinai Medical Center Los Angeles, CA United States

**Keywords:** artificial intelligence, machine learning, health equity, health care disparities, algorithmic bias, social determinants of health, decision making, algorithms, gray literature, equity, health data

## Abstract

**Background:**

Emerging artificial intelligence (AI) applications have the potential to improve health, but they may also perpetuate or exacerbate inequities.

**Objective:**

This review aims to provide a comprehensive overview of the health equity issues related to the use of AI applications and identify strategies proposed to address them.

**Methods:**

We searched PubMed, Web of Science, the IEEE (Institute of Electrical and Electronics Engineers) Xplore Digital Library, ProQuest U.S. Newsstream, Academic Search Complete, the Food and Drug Administration (FDA) website, and ClinicalTrials.gov to identify academic and gray literature related to AI and health equity that were published between 2014 and 2021 and additional literature related to AI and health equity during the COVID-19 pandemic from 2020 and 2021. Literature was eligible for inclusion in our review if it identified at least one equity issue and a corresponding strategy to address it. To organize and synthesize equity issues, we adopted a 4-step AI application framework: Background Context, Data Characteristics, Model Design, and Deployment. We then created a many-to-many mapping of the links between issues and strategies.

**Results:**

In 660 documents, we identified 18 equity issues and 15 strategies to address them. Equity issues related to Data Characteristics and Model Design were the most common. The most common strategies recommended to improve equity were improving the quantity and quality of data, evaluating the disparities introduced by an application, increasing model reporting and transparency, involving the broader community in AI application development, and improving governance.

**Conclusions:**

Stakeholders should review our many-to-many mapping of equity issues and strategies when planning, developing, and implementing AI applications in health care so that they can make appropriate plans to ensure equity for populations affected by their products. AI application developers should consider adopting equity-focused checklists, and regulators such as the FDA should consider requiring them. Given that our review was limited to documents published online, developers may have unpublished knowledge of additional issues and strategies that we were unable to identify.

## Introduction

### Background and Rationale

The use of artificial intelligence (AI) in clinical care and public health contexts has expanded rapidly in recent years [1-6], including throughout the COVID-19 pandemic [7-15]. While emerging AI applications have the potential to improve health care quality and fairness [16-21], they may alternatively perpetuate or exacerbate inequities if they are not designed, deployed, and monitored appropriately [22-26].

Health equity is defined by the World Health Organization as “the absence of unfair and avoidable or remediable differences in health among population groups defined socially, economically, demographically, or geographically.... Pursing health equity means...giving special attention to the needs of those at greatest risk of poor health, based on social conditions.” [27]. According to the Robert Wood Johnson Foundation, “achieving health equity requires identifying and addressing not only overt discrimination but also unconscious and implicit bias and the discriminatory effects—intended and unintended—of structures and policies created by historical injustices, even when conscious intent is no longer clearly present.” [28].

Concerns about AI’s impact on health equity have been discussed extensively in academic and gray literature. Several frameworks identify AI health equity issues throughout development and propose strategies to address them. For example, Chen et al [29] created a 5-step ethical pipeline for health care model development and recommended best practices at each step. Others have proposed similar 6-, 5-, or 4-step frameworks [21,30,31]. Catering more directly to practitioners, researchers at Chicago Booth created an “algorithmic bias playbook” [32]: step-by-step instructions for organizations to identify, improve, and protect against biased algorithms so that fairness is enhanced for vulnerable populations. These frameworks focus on developers as the stakeholder with both the responsibility and the means to improve health equity outcomes. A recent report from Imperial College London built upon Chen et al’s framework to further describe several health equity issues, suggest more detailed strategies, and advocate for action from a broader range of stakeholders, including policymakers [33].

While the aforesaid frameworks related to AI and equity were disseminated between 2016 and 2022, none link equity strategies to multiple issues. An investigation identifying links between health equity issues and strategies to address them is warranted so that stakeholders can understand the universe of approaches to improve health equity at all stages of AI application development and deployment.

### Objectives

The objective of this scoping review was to identify equity issues for health AI applications and connect each issue with corresponding strategies. In addition, we sought to produce a framework that would be useful to independent evaluators whose role is to make comprehensive recommendations for strategies to address equity-relevant issues.

The objective of this review was established in consultation with the study sponsor as part of a broader project examining AI, COVID-19, and health equity. Stakeholder consultation, initial document searches, and document screening were undertaken as part of this broader project and are also described in a separate article on the use of AI in the COVID-19 response [34].

## Methods

### Overview

We adopted a scoping review approach [35] to identify and describe equity issues arising due to implementation of AI in health and catalog strategies to address each issue. In performing the scoping review, we followed the 5 steps described by Arksey and O’Malley [35], although we opted to begin the recommended optional stakeholder consultation before conducting the literature review so that our stakeholders could assist with our search strategy development. We elected a scoping review approach because it is well-suited to “[summarize] findings from a body of knowledge that is heterogeneous in methods or discipline” such as available academic and gray literature [36]. We followed the PRISMA-ScR (Preferred Reporting Items for Systematic Reviews and Meta-Analyses Extension for Scoping Reviews; Multimedia Appendix 1) reporting guidelines as we designed and executed our review [36]. While the study protocol is not published online, Multimedia Appendix 2 includes a detailed description of the search strategy.

### Preparatory Stakeholder Consultation

To best understand the contextual landscape of our scoping review, we began our project by consulting a diverse group of 9 health care stakeholders: 1 patient advocate, 2 clinicians, 1 health system representative, 1 health insurance representative, 1 public policymaker, 1 public health official, 1 industry representative, and 1 researcher. Interviews with these stakeholders helped us define what was in scope for our review and refine inclusion and exclusion criteria for our literature search strategy. The stakeholders we interviewed also identified exemplar peer-reviewed and gray literature documents, existing frameworks, and example lists of issues and strategies. The stakeholder interview protocol, which was provided to stakeholders and also covered topics related to AI and health equity as part of a broader research study, is available in Multimedia Appendix 2.

### Eligibility Criteria

Documents were considered eligible for inclusion in our literature search if (1) they were available in the English language, (2) they related to AI, and (3) they discussed health equity or the clinical or public health response to COVID-19. For documents unrelated to COVID-19, the literature search included publications between January 1, 2014, and December 10, 2021. For documents related to COVID-19, the literature search was limited to the period from December 31, 2019, to December 2021.

### Information Sources and Search Strategy

We searched 3 databases to identify academic literature of interest: PubMed, Web of Science, and the IEEE (Institute of Electrical and Electronics Engineers) Xplore Digital Library. As directed by the medical reference librarian who assisted us with our search strategy, we also searched 2 databases to identify news articles and media commentaries of interest, which she believed would be important to identifying emerging issues and strategies that had not yet been evaluated by academic researchers: ProQuest U.S. Newsstream and Academic Search Complete. Finally, we searched the Food and Drug Administration website and ClinicalTrials.gov for documents meeting inclusion criteria. Textbox 1 gives an overview of our search strategy according to the BeHEMoTh (Behavior, Health condition, Exclusions, Models, or Theories) framework [37]. Detailed parameters for the search strategy are provided in Multimedia Appendix 2.

**Search strategy outline using the BeHEMoTh framework [37].**

- *Behavior of interest (artificial intelligence):* artificial intelligence, machine learning, deep learning, supervised learning, unsupervised learning, reinforcement learning unsupervised clustering, unsupervised classification, supervised classification, natural language processing, expert system, rules engine, fuzzy logic, or algorithm.
- *Health context (clinical or public health response to COVID-19):* health, clinic, hospital, therapy, medical, care, COVID-19, public health
- *Model or theory (equity):* equity, fairness, bias, inequality, race, gender, sex, gender, social determinants of health, socioeconomic status, income, minority, disadvantaged, vulnerable, marginalized, disparities, prejudiced, or minority.
- *Exclusions:* documents in a language other than English.

To be included in our review, a document had to relate to the behavior of interest (artificial intelligence) and at least one of the following: the health context (clinical or public health response to COVID-19) or the model or theory (equity).

### Selection of Documents and Data Charting Process

We screened all documents of potential interest to determine which were eligible for full-text review. Articles of potential interest were added to a Microsoft Excel spreadsheet to facilitate the selection process and data charting of our progress. If an article did not have an abstract, it was automatically eligible for full-text review.

For articles with an abstract or summary, we used a multistep process to screen for inclusion in the full-text review. First, 3 members of the study team (CTB, LB, and SM) independently screened a random sample of 6% (120/1897) of articles and discussed disagreements among the reviewers about whether articles should be included. We held a series of meetings to refine and finalize our screening criteria to improve agreement among our team. Second, we used single-reviewer screening to determine inclusion for the remaining 94% (1777/1897) of documents. Third, we used random dual review of a sample (445/1777, 25.04%) of documents that had only been reviewed by a single reviewer so that we could measure and report interrater agreement. Disagreements in inclusion decisions were resolved through consensus discussion by all 3 reviewers.

We decided to group issues and strategies using a 4-step framework that we adapted from previously published AI development pipeline literature sources [21,29-31]. The closest preexisting framework was described by Chen et al [29] as including 5 categories: Problem Selection, Data Collection, Outcome Definition, Algorithm Development, and Postdeployment Considerations. To make our results understandable to the broadest possible set of stakeholders, we expanded Chen et al’s original “Problem Selection” category to include other aspects of the Background Context of AI development and use. We retained a category for issues related to Data Characteristics. We collapsed Outcome Definition together with Algorithm Development because they are related design decisions, and we renamed Postdeployment Considerations to Deployment so that all forms of evaluation would be included. Thus, our 4 development categories in the framework became:

- Background Context: systemic and structural elements (eg, factors that influence Problem Selection). For Background Context, we defined systemic and structural elements as the societal and organizational characteristics influencing developers, including the rules and regulations in place at the local, regional, and national levels.
- Data Characteristics: quality and quantity of the data.
- Design: choice of model, variables, outcome definition, and objective function.
- Deployment: model evaluation, use, and maintenance.

### Abstraction of Data Items for Issues and Strategies

Each article undergoing full-text review was reviewed by 1 of 3 members of the study team. Relevant citations listed in these articles were also reviewed to identify additional data sources. Our unit of analysis was an issue-strategy pair, defined as the linking of a particular equity issue to a potential strategy that could be used to improve equity for the AI application in health care. We defined an issue as a potential equity-related problem that had been suggested by at least one document author, and we defined a strategy as a recommended action to address an issue. We extracted issues and strategies named in each article using a data collection form consisting of the reference for each document, the specific issue(s) that the document discussed, and which strategies that the article proposed could be used to address the issue. Each document could include multiple issue-strategy pairs. We also abstracted the following items for each issue: narrative description of the issue, issue group (prespecified categories: Background Context, Data Characteristics, Design, and Deployment), representative quotes from the document, and representative quotes describing strategies. We included issues and strategies that were speculative or theoretical in addition to those that have been “proven” to exist, because we believed this information would likely be valuable to developers and regulators who are interested in learning about emerging issues and solutions.

### Synthesis of Results

We created our set of issues and strategies inductively: whenever an equity issue or strategy discussed in a document was not adequately described by the current set, we created a new entry. Definitions were refined in group meetings among the 3 members of the study team.

### Ethics and Human Participants

The RAND Corporation Human Subjects Protection Committee (HSPC ID 2021-N0625), which functions as RAND’s Institutional Review Board, determined that our study qualified for exemption from committee review.

### Results From the Preliminary Stakeholder Consultation

Our stakeholders did not suggest any changes to the study topics proposed for our review. They suggested that we should include gray literature documents such as news articles, clinical trial protocols, and conference proceedings in our review in addition to peer-reviewed articles. Stakeholders also suggested that we investigate several topics related to AI and equity that they believed warranted further research (Textbox 2).

**Stakeholder recommendations for areas of focus in the scoping review.**

*Data sets, variable selection, and health equity*

Stakeholders emphasized that there was a gap in current understanding about how limitations in training and validation data sets influenced AI application performance for vulnerable subpopulations and how strategies could be undertaken to protect such subpopulations. They also expressed concern that there was a tension in ensuring inclusion of underrepresented groups while also ensuring privacy for patients from such groups, and that strategies were needed to improve equity due to this tension.

*Limitations in evaluating equity-related outcomes*

Four interviewees suggested that it was important to investigate certain outcomes for vulnerable subgroups of patients, such as measures of cost, quality, and access to care, that might be challenging for developers to obtain.

*Availability of equity-related information on AI algorithm performance*

Four interviewees mentioned that AI may be used internally by an organization such as a health system or government agency, and that publicly available information about algorithm performance for vulnerable subgroups might be limited.

See Multimedia Appendix 3 for additional results from the stakeholder consultation.

## Results

### Search Output

Our search strategy identified a total of 2244 unique documents of potential interest. We conducted title and abstract review for 1897 documents or trial records, with 313 meeting inclusion criteria. For a 25% (445/1777) sample of records that were reviewed by 2 reviewers, interreviewer agreement on inclusion was 88% (391/445; Cohen κ=0.61) [38]. We identified an additional 347 documents of interest that did not have abstracts to review, so they all underwent full-text review (296 news articles and 51 Food and Drug Administration documents).

**Figure 1:**
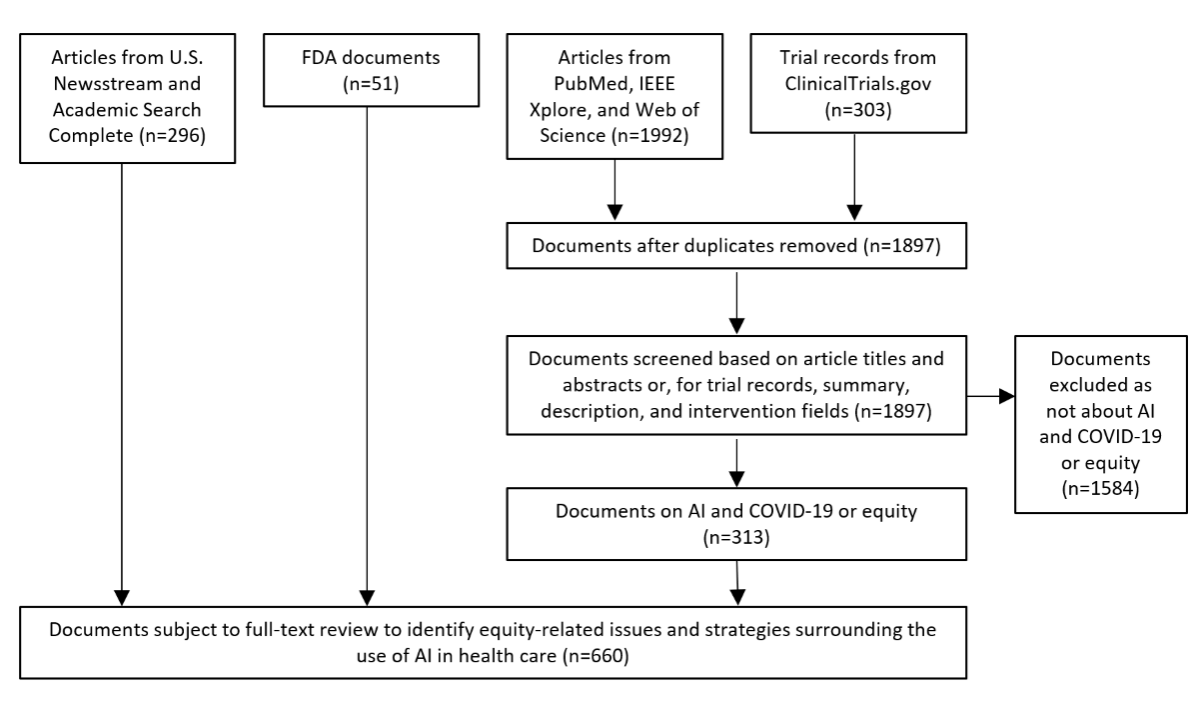
PRISMA (Preferred Reporting Items for Systematic Reviews and Meta-Analyses) flow diagram. AI: artificial intelligence; FDA: Food and Drug Administration.

In total, 660 documents meeting inclusion criteria underwent full-text review and were included in our analysis. The PRISMA flow diagram displaying the literature search and screening results is presented in Figure 1 [36].

### Equity Issues and Strategies in Health AI

This section will present three tables and one figure that highlight the issues affecting equity for AI applications as well as the strategies we identified to address them.

**Figure 2:**
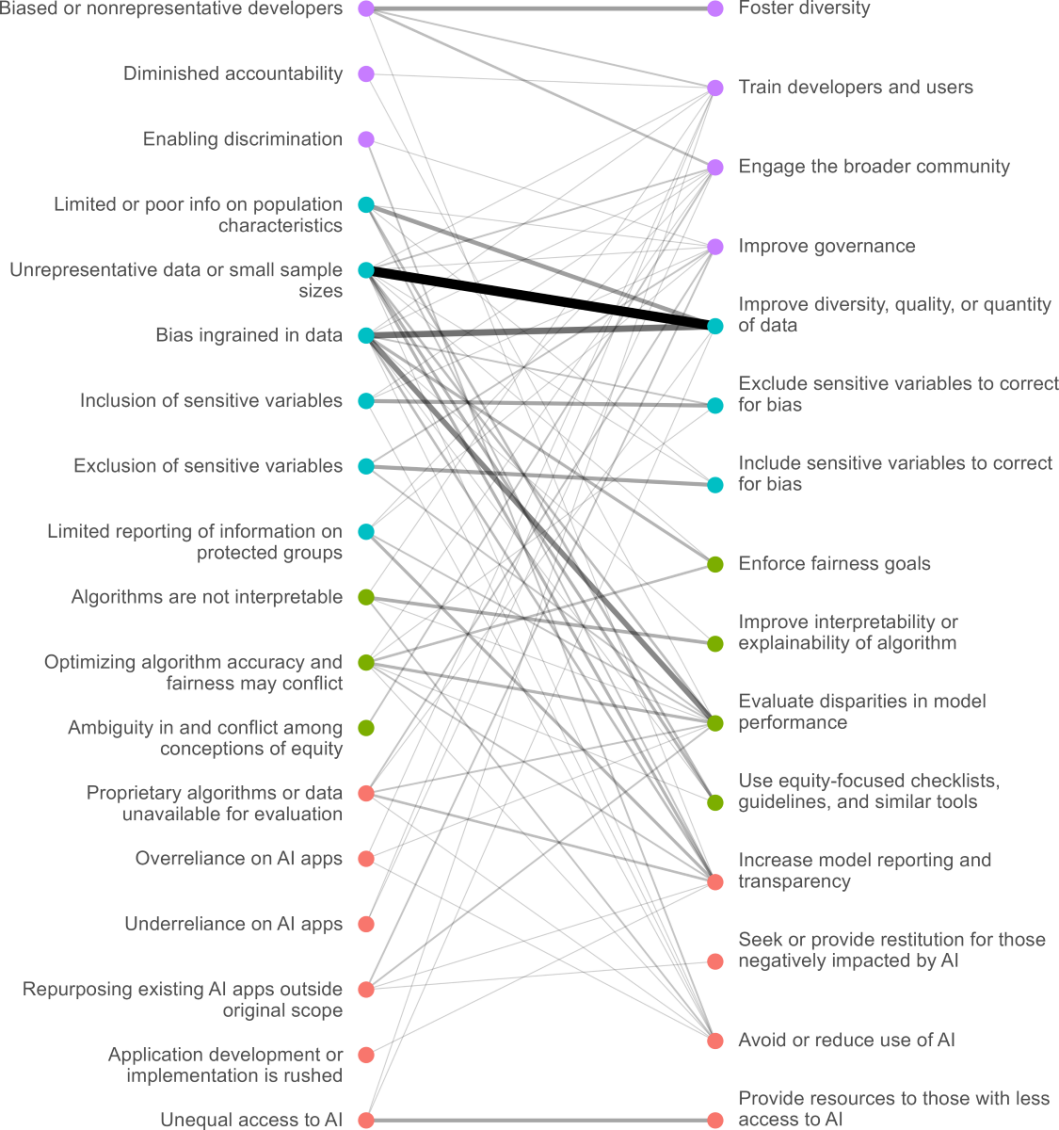
Issues related to AI and equity and strategies proposed to address them. The thickness and opacity of each line connecting an issue to a strategy are proportional to how frequently they were mentioned together. AI: artificial intelligence.

We identified a total of 18 issues linked to 15 strategies. We present our main results in 2 parts. Tables 1 and 2 display the issues and strategies, respectively, that we identified in the literature, and we provide a brief narrative description for each item. Then, Figure 2 and Table 3 demonstrate how issues and strategies were linked together. The complete list of documents that identified each issue-strategy pair is provided in Multimedia Appendix 4.

**Table 1:** Issues related to AI^a^ and health equity that were abstracted from the literature.

Category and issue		Description
**Background Context**		
	Biased or nonrepresentative developers	Development team composition may be biased or poorly representative of the population, leading to mismatched priorities and blind spots.
	Diminished accountability	Lack of developer accountability makes it difficult for individuals harmed by AI applications to obtain compensation.
	Enabling discrimination	Developers may use AI algorithms to purposely discriminate for malice or for economic gain.
**Data Characteristics**		
	Limited information on population characteristics	Insufficiently granular data on population characteristics may lead to inappropriately aggregating dissimilar groups, such as classifying race into only White and non-White.
	Unrepresentative data or small sample sizes	Inadequate representation of groups in training data can lead to worse model performance in these groups, especially when training and deployment populations are poorly matched.
	Bias ingrained in data	When data reflect past disparities or discrimination, algorithms may incorporate and perpetuate these patterns.
	Inclusion of sensitive variables	Inclusion of sensitive information, such as race or income, may cause algorithms to inappropriately discriminate on these factors.
	Exclusion of sensitive variables	Exclusion of sensitive information may reduce accuracy in some groups and lead to systematic bias due to a lack of explanatory power.
	Limited reporting of information on protected groups	Lack of reporting on the composition of training data or model performance by group makes it difficult to know where to appropriately use models and whether they have disparate impacts.
**Model Design**		
	Algorithms are not interpretable	When we do not understand why models make decisions, it is difficult to evaluate whether the decision-making approach is fair or equitable.
	Optimizing algorithm accuracy and fairness may conflict	Optimizing models for fairness may introduce a trade-off between model accuracy and the fairness constraint, meaning that equity may come at the expense of decreased accuracy.
	Ambiguity in and conflict among conceptions of equity	There are many conceptions of fairness and equity, which may be mutually exclusive or require sensitive data to evaluate.
**Deployment Practices**		
	Proprietary algorithms or data unavailable for evaluation	When training data, model design, or the outputs of algorithms are proprietary, regulators and other independent evaluators may not be able to effectively assess risk of bias.
	Overreliance on AI applications	Users may blindly trust algorithmic outputs, implementing decisions despite contrary evidence and perpetuating biases if the algorithm is discriminatory.
	Underreliance on AI applications	People may be dismissive of algorithm outputs that challenge their own biases, thereby perpetuating discrimination.
	Repurposing existing AI applications outside original scope	Models may be repurposed for use with new populations or to perform new functions without sufficient evaluation, bypassing safeguards on appropriate use.
	Application development or implementation is rushed	Time constraints may exacerbate equity issues if they push developers to inappropriately repurpose existing models, use low-quality data, or skip validation.
	Unequal access to AI	AI applications may be deployed more commonly in high-income areas, potentially amplifying preexisting disparities.

^a^AI: artificial intelligence.

**Table 2:** Strategies to address AI^a^ equity issues that were abstracted from the literature.

Category and strategy			Description
**Background Context**			
	Foster diversity	Create AI development teams with diverse characteristics, experiences, and roles to increase consideration of equity throughout development and decrease blind spots.	
	Train developers and users	Train AI developers and users in equity considerations and the ethical implications of AI, as these topics may be unfamiliar to some.	
	Engage the broader community	Foster community involvement throughout development, from conception to postdeployment, to increase the likelihood that developers prioritize equity concerns.	
	Improve governance	Enact robust regulation and industry standards to align AI applications with social norms, including equity, safety, and transparency.	
**Data Characteristics**			
	Improve diversity, quality, or quantity of data	Train models with large, diverse samples that are representative of the target population for the application and contain all relevant features.	
	Exclude sensitive variables to correct for bias	Exclude sensitive variables or replace them with variables that are more directly relevant to health outcomes to prevent models from discriminating directly on these characteristics.	
	Include sensitive variables to correct for bias	Include sensitive variables to improve model accuracy, increase explanatory power, and enable easier testing for inequitable impact.	
**Model Design**			
	Enforce fairness goals	Formulate a fairness norm and enforce it in the model by editing the input data, objective function, or model outputs.	
	Improve interpretability or explainability of the algorithm	Choose models that are inherently explainable (such as decision trees), build models with post hoc explainability, or explore explainable local approximations to model decision making.	
	Evaluate disparities in model performance	Evaluate model performance on a wide range of metrics across subgroups, particularly groups that might face inequitable impact, then report and act upon the results.	
	Use equity-focused checklists, guidelines, and similar tools	Incorporate equity-focused checklists into workflows for developers, reviewers of AI models, health care providers using an application, or patients who want to understand algorithm outputs.	
**Deployment Practices**			
	Increase model reporting and transparency	Provide more information on AI equity issues, including publishing standardized equity-related analyses on models, increasing independent model reviews, and requiring equity discussions in academic journals.	
	Seek or provide restitution for those negatively impacted by AI	Proactively provide restitution to those harmed by AI or create legal frameworks so they can seek restitution.	
	Avoid or reduce use of AI	Consider discontinuing model use if equity sequelae are severe or if improvement efforts have been fruitless.	
	Provide resources to those with less access to AI	Improve access to AI for disadvantaged groups and low-income countries by subsidizing infrastructure, creating education programs, or hosting AI conferences in these locations.	

^a^AI: artificial intelligence.

**Table 3:** The most common strategies mentioned in the literature for each health equity issue.

Category and issue			Issue frequency (N=195), n (%)		Most frequently linked strategy		Second most frequently linked strategy
**Background Context**							
	Biased or nonrepresentative developers	13 (6.7)		Foster diversity		Engage the broader community	
	Diminished accountability	2 (1.0)		Evaluate disparities in model performance		Train developers and users	
	Enabling discrimination	3 (1.5)		Avoid or reduce use of AI^a^		Improve governance	
**Data Characteristics**							
	Limited information on population characteristics	14 (7.2)		Improve diversity, quality, or quantity of data		Use equity-focused checklists, guidelines, and similar tools	
	Unrepresentative data or small sample sizes	46 (23.6)		Improve diversity, quality, or quantity of data		Increase model reporting and transparency	
	Bias ingrained in data	37 (19.0)		Improve diversity, quality, or quantity of data		Evaluate disparities in model performance	
	Inclusion of sensitive variables	9 (4.6)		Exclude sensitive variables to correct for bias		Avoid or reduce use of AI	
	Exclusion of sensitive variables	10 (5.1)		Include sensitive variables to correct for bias		Evaluate disparities in model performance	
	Limited reporting of information on protected groups	8 (4.1)		Increase model reporting and transparency		Evaluate disparities in model performance	
**Model Design**							
	Algorithms are not interpretable	9 (4.6)		Improve interpretability or explainability of algorithm		Avoid or reduce use of AI	
	Optimizing algorithm accuracy and fairness may conflict	13 (6.7)		Evaluate disparities in model performance		Enforce fairness goals	
	Ambiguity in and conflict among conceptions of equity	2 (1.0)		Engage the broader community		—^b^	
**Deployment Practices**							
	Proprietary algorithms or data unavailable for evaluation	9 (4.6)		Increase model reporting and transparency		Evaluate disparities in model performance	
	Overreliance on AI applications	3 (1.5)		Avoid or reduce use of AI		Evaluate disparities in model performance	
	Underreliance on AI applications	2 (1.0)		Engage the broader community		Train developers and users	
	Repurposing existing AI applications outside original scope	6 (3.1)		Evaluate disparities in model performance		Improve governance	
	Application development or implementation is rushed	1 (0.5)		Increase model reporting and transparency		—	
	Unequal access to AI	8 (4.1)		Provide resources to those with less access to AI		Improve diversity, quality, or quantity of data	

^a^AI: artificial intelligence.

^b^Only 1 issue has been linked to the strategy.

### Linking Issues and Strategies

In this section, we report how issues and strategies have been linked in the articles we reviewed. The strategies most frequently linked to each issue are shown in Table 3, and the references provided in Multimedia Appendix 2 offer more detail on how to apply a strategy to a given issue. A small number of issues comprise the majority of mentions in the literature: The top 5 issues constitute 63% (123/195, 63.1%) of all issue-strategy pairs. Each of these issues has several well-developed strategies, usually focused on improving the quality of data or evaluating bias in model-decision making. By contrast, other issues are mentioned infrequently and do not have well-developed strategies. When only 1 issue has been linked to a strategy, the second column is presented with an em dash. We included an issue frequency column as a measure of how often issues have been mentioned in the literature.

Figure 2 is a map of the 195 issue-strategy pairs identified in the literature, and it shows a complex many-to-many mapping between issues and strategies in health equity and highlights which strategies and issues are most common. Each issue-strategy pair mentioned in the literature is shown as a link. Bolder lines indicate strategies and issues that are more frequently linked. A comprehensive list of links and the corresponding references is provided in Multimedia Appendix 4. Out of the total 195 issue-strategy pairs, 50.3% (98/195) were identified in peer-reviewed literature. The remaining 49.7% (97/195) were from gray literature sources, including 14 conference proceedings, 11 news articles, 5 textbooks, 3 preprints, 2 press releases, 1 thesis, 1 clinical trial record, and 60 others (eg, reports and briefings).

## Discussion

### Principal Findings

By analyzing the literature on AI and health disparities we have identified 18 issues and 15 strategies that can be used to improve health equity in the realm of AI. Our work builds upon frameworks from the existing literature, identifying named strategies and issues for each stage of AI development and implementation. In addition, we draw 3 new insights from mapping the relationships between issues and strategies.

The framework published by Chen et al [29] offers 5 recommendations for improving equity, which can be paraphrased as follows: (1) problems should be tackled by diverse teams using frameworks that increase the probability that equity will be achieved; (2) data collection should be framed as an important front-of-mind concern, including encouragement of disclosing imbalanced data sets; (3) outcome choice should reflect the task at hand in an unbiased manner; (4) developers should reflect on the goals of the model during development and preanalysis; and (5) audits should be designed to identify specific harms, including harms at the level of the group rather than at the population. While these are important and sound recommendations, our results additionally emphasize the need to engage with communities throughout the development and deployment phases, identify opportunities for equity-focused governance at the local and national levels, and identify additional opportunities for improvement after algorithms are found to impair equity (eg, avoiding or reducing AI use, providing resources to those with less access to AI, and providing restitution to those negatively impacted by AI). Our comprehensive mapping of issues and strategies can be useful to stakeholders of all types, including developers, representatives of vulnerable groups, and regulators.

### The Literature Focuses on a Small Set of Issues

A small set of issues dominates the literature. The top 5 issues comprise nearly two thirds of all issue-strategy pairs. The discourse around health AI equity focuses on Data Characteristics: almost two-thirds of all issue-strategy pairs are related to data. These issues are widely researched, and, therefore, we encountered many corresponding strategies to address them. Some strategies directly address data quality, while others accept data limitations and try to improve fairness despite poor data quality.

Much of the discourse on model design focuses on the trade-off between accuracy and fairness [39-43]. This multifaceted problem requires that stakeholders select a definition of fairness and analyze how accuracy/fairness trade-offs will balance in specific applications. The most common approach to improving model design involves measuring disparities in model performance and revising the model to enforce fairness goals [44]. As definitions of fairness may conflict, developers and evaluators should test the impact of different constraints across a broad range of metrics (such as accuracy, false-positive rate, and false-negative rate) and report group-level disparities in each of these metrics [45]. Equity-relevant model design literature is most developed for classification or regression tasks, and there is less guidance in other areas such as online learning [46]. Relevant subgroups are often application specific, and the data on these subgroups may not be available [47].

Other issues were rarely discussed and have a limited number of associated strategies. For example, several issues reflect concerns about how AI is deployed—especially when AI applications are used outside their original scope or when they are rushed through development and into production without sufficient testing.

Even if an issue is not frequently discussed in the literature, it may still be important. In other words, an issue may not be discussed frequently because there is limited evidence of equity impact or because corresponding strategies are underdeveloped. We believe that some issues may have been insufficiently discussed despite their promise as topics that would benefit from future research. For example, future work is warranted to investigate the negative impacts of the following issues: repurposing AI applications outside their original scope, inadequate descriptions of population characteristics, and lack of accountability for the unintended consequences of AI on health equity.

### Strategies Are Multipurpose

While some strategies, such as improving interpretability, are tailored to specific issues, most strategies are multipurpose. The top 5 most frequently mentioned strategies, which account for more than half of issue-strategy pairs in our sample, are collectively linked to all 18 issues. Each of these strategies is linked to critical aspects of application development. Evaluating disparities in model performance is often necessary for quantifying bias across subgroups. Similarly, improving data is important across a broad range of issues because the decision-making logic of AI models flows directly from training data. Community engagement and improved governance can increase the consideration of equity issues throughout all stages of AI algorithm development. Community stakeholders should be involved at all stages of production, including deciding whether an application should be built, setting goals for the model, defining fairness [48], and guarding against unintended consequences after deployment [21,49-51]. Improving governance is usually advocated in the form of guiding principles for AI use [25,52] or “soft governance” such as industry-organized protocols [53,54]. Regulation is not frequently advocated, although it is unclear whether this is because researchers believe regulation would be ineffective or because they prefer to focus on technical solutions.

### Small Sets of Strategies Can Address a Broad Set of Issues

Sometimes it is only practical to focus on a small set of strategies. For instance, in their Algorithmic Bias Playbook, Obermeyer et al [32] suggested that organizations identify biased algorithms and then retrain them on less biased targets, improve the representativeness of their data set, or consider discontinuing their use.

Once stakeholders have identified issues that are relevant for a specific application, they can use Table 3 and Figure 2 to select a set of strategies to address them. The most common 5 strategies cited above are a good starting point because of their broad coverage of issues. However, not all these strategies may be feasible, and others may require complementation with additional strategies to fully address a specific issue.

Consider an example use case for our mapping of equity-relevant issues and strategies to address them: A developer has been commissioned to build an open-source predictive model of emergency department admission probability based on electronic health records. The developer has identified data issues related to bias and representativeness, but is also concerned that the model may be less accurate for some subgroups of patients. The developer may consider the top 5 most common strategies first, and then may realize that modifying the data collection process is infeasible. Although improving governance does not necessarily require new legislative or regulatory action, it does involve collective action between industry and the broader community, so it may seem feasible in certain scenarios. However, the remaining 3 of the top 5 strategies can be implemented by a single stakeholder without coordinating collective action across different groups. Anyone with model access and demographic data can evaluate disparities in model performance and increase model reporting and transparency. Similarly, all developers can seek input from affected communities when they begin the development process.

The developer could then use Figure 2 to select a set of complementary strategies specific to some of the issues. If their evaluation did find disparate performance across groups, then they could enforce fairness constraints in the input data, model design, or model outputs. They may also review the model using an equity-focused checklist, such as the Prediction Model Risk of Bias Assessment Tool (PROBAST) [55], as this is low-cost and may identify other avenues to improve equity. They may also decide that they can better engage with the relevant stakeholders if they can explain the model’s decision-making processes and develop model report cards for equity.

After completing this exercise, the developer will have identified an initial set of strategies that is within their scope of action. This set may evolve over time, especially as the broader community is engaged: For example, community stakeholders may help identify important features the developer overlooked (such as social determinants of health), suggest different definitions of equity, or question whether AI should be used at all [56].

This use-case example is one approach to addressing a complex set of equity issues. For most AI applications, we expect that developers will be able to identify a small set of strategies to address a broad range of equity issues. Particularly important issues may require multiple complementary strategies. We recommend that developers start by considering which of the 5 most common strategies are suitable for an application and then adding additional complementary strategies as needed—particularly low-cost strategies such as the use of the PROBAST checklist.

### Limitations

This scoping review has several limitations. First, due to space constraints, the descriptions of each issue and strategy are brief. This means that stakeholders may need to access additional resources to take action and operationalize a strategy. For instance, if enforcing fairness goals is identified as a useful strategy, stakeholders need to decide what fairness rule to use and how to modify data inputs, the model objective function, or model outputs [21,57-60]. To better understand issues and strategies, stakeholders should use Multimedia Appendix 4 to find relevant documents. More detailed descriptions of issues and strategies will also be available in a subsequent report that will be published by the funder of this study, the Patient-Centered Outcomes Research Institute.

Second, some issues and strategies may conflict. For example, both inclusion and exclusion of sensitive variables are discussed as having either a positive or a negative influence on the impact of health AI on equity, depending on context and perspective. As a result, we include these as both issues and strategies in our study, reflecting the unsettled and context-dependent nature of debate on this topic within the literature.

Third, our search strategy included gray literature sources, so some of the issue-strategy pairs are likely to be speculative rather than proven to be effective. Out of 195 issue-strategy pairings, 98 were from peer-reviewed literature and 97 were from gray literature sources such as reports, news articles, conference proceedings, and preprint articles. Readers should consult the sources of the issue-strategy pairs when determining whether a given strategy should be used.

Fourth, we did not rate the quality of issues, strategies, or the articles from which we identified issue-strategy pairs. Some sources go into detail about health equity issues and strategies, others only make general recommendations or may represent outmoded views. The goal of this scoping review was to identify which issues and strategies are highlighted in the literature. Future reviews could instead focus on identifying the best or most developed strategies.

Fifth, the issues and strategies we identified are not entirely distinct: some are intermediaries that lead to other issues or strategies. For instance, repurposing an application is not inherently inequitable, but may increase the chance that the training data are unrepresentative of the target population. Similarly, uninterpretable algorithms do not create biased outcomes, but make them more difficult to detect. The same applies to strategies: using equity checklists does not directly solve problems, but makes it more likely that developers identify equity issues and appropriate strategies. We included these intermediary issues and strategies because they provide a richer description of intervention points for promoting health equity.

Sixth, there are other prominent concerns about AI and equity that were out of scope for our review. For example, AI applications may displace human workers in ways that could increase economic and health disparities, or the default use of female voices in AI assistants that perform clerical tasks may perpetuate bias and lead to negative effects on health equity for women [51]. While these concerns are raised in the context of economic or social disparities, we found no discussion of their impact on health equity specifically, and thus did not include them in our study.

### Conclusions

Our work contributes to a growing body of AI health equity literature. We add to this literature by creating a many-to-many mapping between strategies and issues and by reviewing the literature to identify how often each strategy is linked to each issue. This scoping review is useful for a wide array of stakeholders, including developers, users, policymakers, and researchers who may wish to implement strategies to improve health equity for vulnerable populations of interest. While no set of strategies can eliminate the equity concerns posed by health AI, small sets of strategies can often mitigate many of the most pressing issues. We should also recognize that existing nonalgorithmic decision making is imperfect. By thoughtfully adopting complementary sets of strategies that cover a broad range of equity issues, AI models may offer improvements in equity over the status quo.

## Appendix

Multimedia Appendix 1 PRISMA-ScR (Preferred Reporting Items for Systematic Reviews and Meta-Analyses Extension for Scoping Reviews) checklist.

Multimedia Appendix 2 Literature search documentation and stakeholder interview protocol.

Multimedia Appendix 3 Results from stakeholder consultation.

Multimedia Appendix 4 Table of documents linking AI health equity issues and strategies.

## Abbreviations

AI: artificial intelligence
BeHEMoTh: Behavior, Health condition, Exclusions, Models, or Theories
FDA: Food and Drug Administration
IEEE: Institute of Electrical and Electronics Engineers
PRISMA-ScR: Preferred Reporting Items for Systematic Reviews and Meta-Analyses Extension for Scoping Reviews
PROBAST: Prediction Model Risk of Bias Assessment Tool

## References

[1] Secinaro S, Calandra D, Secinaro A, Muthurangu V, Biancone P (2021). The role of artificial intelligence in healthcare: a structured literature review. BMC Med Inform Decis Mak.

[2] Yin J, Ngiam KY, Teo HH (2021). Role of Artificial Intelligence Applications in Real-Life Clinical Practice: Systematic Review. J Med Internet Res.

[3] Noorbakhsh-Sabet N, Zand R, Zhang Y, Abedi V (2019). Artificial Intelligence Transforms the Future of Health Care. Am J Med.

[4] Rajpurkar P, Chen E, Banerjee O, Topol EJ (2022). AI in health and medicine. Nat Med.

[5] Topol E (2019). Deep medicine: how artificial intelligence can make healthcare human again.

[6] Rajkomar A, Dean J, Kohane I (2019). Machine Learning in Medicine. N Engl J Med.

[7] Gunasekeran DV, Tseng RMWW, Tham Y, Wong TY (2021). Applications of digital health for public health responses to COVID-19: a systematic scoping review of artificial intelligence, telehealth and related technologies. NPJ Digit Med.

[8] Wynants L, Van Calster B, Collins GS, Riley RD, Heinze G, Schuit E, Bonten MMJ, Dahly Darren L, Damen Johanna A A, Debray Thomas P A, de Jong Valentijn M T, De Vos Maarten, Dhiman Paul, Haller Maria C, Harhay Michael O, Henckaerts Liesbet, Heus Pauline, Kammer Michael, Kreuzberger Nina, Lohmann Anna, Luijken Kim, Ma Jie, Martin Glen P, McLernon David J, Andaur Navarro Constanza L, Reitsma Johannes B, Sergeant Jamie C, Shi Chunhu, Skoetz Nicole, Smits Luc J M, Snell Kym I E, Sperrin Matthew, Spijker René, Steyerberg Ewout W, Takada Toshihiko, Tzoulaki Ioanna, van Kuijk Sander M J, van Bussel Bas, van der Horst Iwan C C, van Royen Florien S, Verbakel Jan Y, Wallisch Christine, Wilkinson Jack, Wolff Robert, Hooft Lotty, Moons Karel G M, van Smeden Maarten (2020). Prediction models for diagnosis and prognosis of covid-19: systematic review and critical appraisal. BMJ.

[9] Musulin J, Baressi Šegota Sandi, Štifanić Daniel, Lorencin I, Anđelić N, Šušteršič Tijana, Blagojević A, Filipović N, Ćabov T, Markova-Car E (2021). Application of Artificial Intelligence-Based Regression Methods in the Problem of COVID-19 Spread Prediction: A Systematic Review. Int J Environ Res Public Health.

[10] Rasheed J, Jamil A, Hameed AA, Al-Turjman F, Rasheed A (2021). COVID-19 in the Age of Artificial Intelligence: A Comprehensive Review. Interdiscip Sci.

[11] Jamshidi M, Roshani S, Talla J, Lalbakhsh A, Peroutka Z, Roshani S, Parandin F, Malek Z, Daneshfar F, Niazkar Hr, Lotfi S, Sabet A, Dehghani M, Hadjilooei F, Sharifi-Atashgah Ms, Lalbakhsh P (2022). A Review of the Potential of Artificial Intelligence Approaches to Forecasting COVID-19 Spreading. AI.

[12] Malik YS, Sircar S, Bhat S, Ansari MI, Pande T, Kumar P, Mathapati B, Balasubramanian G, Kaushik R, Natesan S, Ezzikouri S, El Zowalaty ME, Dhama K (2021). How artificial intelligence may help the Covid-19 pandemic: Pitfalls and lessons for the future. Rev Med Virol.

[13] Rasheed J, Jamil A, Hameed AA, Aftab U, Aftab J, Shah SA, Draheim D (2020). A survey on artificial intelligence approaches in supporting frontline workers and decision makers for the COVID-19 pandemic. Chaos Solitons Fractals.

[14] Rasheed J, Shubair RM (2022). Screening Lung Diseases Using Cascaded Feature Generation and Selection Strategies. Healthcare (Basel).

[15] Rasheed J (2022). Analyzing the Effect of Filtering and Feature-Extraction Techniques in a Machine Learning Model for Identification of Infectious Disease Using Radiography Imaging. Symmetry.

[16] Pérez-Stable Eliseo J, Jean-Francois B, Aklin CF (2019). Leveraging Advances in Technology to Promote Health Equity. Med Care.

[17] Veinot TC, Ancker JS, Bakken S (2019). Health informatics and health equity: improving our reach and impact. J Am Med Inform Assoc.

[18] Chen IY, Szolovits P, Ghassemi M (2019). Can AI Help Reduce Disparities in General Medical and Mental Health Care?. AMA J Ethics.

[19] Chen IY, Joshi S, Ghassemi M (2020). Treating health disparities with artificial intelligence. Nat Med.

[20] Thomasian NM, Eickhoff C, Adashi EY (2021). Advancing health equity with artificial intelligence. J Public Health Policy.

[21] Rajkomar A, Hardt M, Howell MD, Corrado G, Chin MH (2018). Ensuring Fairness in Machine Learning to Advance Health Equity. Ann Intern Med.

[22] Panch T, Mattie H, Celi LA (2019). The "inconvenient truth" about AI in healthcare. NPJ Digit Med.

[23] Panch T, Pearson-Stuttard J, Greaves F, Atun R (2019). Artificial intelligence: opportunities and risks for public health. Lancet Digit Health.

[24] Parikh RB, Teeple S, Navathe AS (2019). Addressing Bias in Artificial Intelligence in Health Care. JAMA.

[25] World Health Organization (2021). Ethics and Governance of Artificial Intelligence for Health: WHO Guidance.

[26] Mhasawade V, Zhao Y, Chunara R (2021). Machine learning and algorithmic fairness in public and population health. Nat Mach Intell.

[27] World Health Organization (2022). Social determinants of health: Health equity.

[28] Braveman P, Arkin E, Orleans T, Proctor D, Plough A (2017). What is health equity? And what difference does a definition make?. National Collaborating Centre for Determinants of Health.

[29] Chen IY, Pierson E, Rose S, Joshi S, Ferryman K, Ghassemi M (2021). Ethical Machine Learning in Healthcare. Annu Rev Biomed Data Sci.

[30] Vokinger KN, Feuerriegel S, Kesselheim AS (2021). Mitigating bias in machine learning for medicine. Commun Med (Lond).

[31] Yeung D, Khan I, Kalra N, Osoba O (2021). Identifying Systemic Bias in the Acquisition of Machine Learning Decision Aids for Law Enforcement Applications. RAND.

[32] Obermeyer Z, Nissan R, Stern M, Eaneff S, Bembeneck E, Mullainathan S (2021). Algorithmic Bias Playbook. Federal Trade Commission.

[33] O'Brien N, Van Dael J, Clarke K, Gardner C, O'Shaughnessy J, Darzi A, Ghafur S (2022). Addressing racial and ethnic inequities in data-driven health technologies. Imperial College London.

[34] Mann S, Berdahl CT, Baker L, Girosi F (2022). Artificial intelligence applications used in the clinical response to COVID-19: A scoping review. PLOS Digit Health.

[35] Arksey H, O'Malley L (2005). Scoping studies: towards a methodological framework. International Journal of Social Research Methodology.

[36] Tricco AC, Lillie E, Zarin W, O'Brien KK, Colquhoun H, Levac D, Moher D, Peters MDJ, Horsley T, Weeks L, Hempel S, Akl EA, Chang C, McGowan J, Stewart L, Hartling L, Aldcroft A, Wilson MG, Garritty C, Lewin S, Godfrey CM, Macdonald MT, Langlois EV, Soares-Weiser K, Moriarty J, Clifford T, Tunçalp Özge, Straus SE (2018). PRISMA Extension for Scoping Reviews (PRISMA-ScR): Checklist and Explanation. Ann Intern Med.

[37] Booth A, Carroll C (2015). Systematic searching for theory to inform systematic reviews: is it feasible? Is it desirable?. Health Info Libr J.

[38] McHugh ML (2012). Interrater reliability: the kappa statistic. Biochem Med (Zagreb).

[39] Rodolfa KT, Lamba H, Ghani R (2021). Empirical observation of negligible fairness–accuracy trade-offs in machine learning for public policy. Nat Mach Intell.

[40] Desiere S, Struyven L (2020). Using Artificial Intelligence to classify Jobseekers: The Accuracy-Equity Trade-off. J. Soc. Pol.

[41] Dutta S, Wei D, Yueksel H, Chen P, Liu S, Varshney K (2020). Is there a trade-off between fairness and accuracy? a perspective using mismatched hypothesis testing. Proceedings of the 37th International Conference on Machine Learning (PMLR), Vol. 119.

[42] Cooper A, Abrams E, Na N, editors (2021). Emergent unfairness in algorithmic fairness-accuracy trade-off research. AIES '21: Proceedings of the 2021 AAAI/ACM Conference on AI, Ethics, and Society.

[43] Liu S, Vicente LN (2022). Accuracy and fairness trade-offs in machine learning: a stochastic multi-objective approach. Comput Manag Sci.

[44] Paul A, Jolley C, Anthony A (2018). Reflecting the Past, Shaping the Future: Making AI Work for International Development. USAID.

[45] Rodolfa K, Saleiro P, Ghani R (2020). Bias and Fairness (Chapter 11). Big Data and Social Science.

[46] Chouldechova A, Roth A The frontiers of fairness in machine learning. arXiv. Preprint posted online on October 20, 2018.

[47] Holstein K, Wortman VJ, Daumé IH, Dudik M, Wallach H (2019). Improving Fairness in Machine Learning Systems: What Do Industry Practitioners Need?. CHI '19: Proceedings of the 2019 CHI Conference on Human Factors in Computing Systems.

[48] Horvitz E, Clyburn M, Griffiths J-M, Matheny J (2020). Privacy and Ethics Recommendations for Computing Applications Developed to Mitigate COVID-19: White Paper Series on Pandemic Response and Preparedness. UNT Digital Library.

[49] Zimmer M, Franco Z, Madiraju P, Echeveste C, Heindel K, Ogle J (2021). Public Opinion Research on Artificial Intelligence in Public Health Responses: Results of Focus Groups with Four Communities. AAAS.

[50] Morley J, Machado CCV, Burr C, Cowls J, Joshi I, Taddeo M, Floridi L (2020). The ethics of AI in health care: A mapping review. Soc Sci Med.

[51] Shachar C, Gerke S, Adashi EY (2020). AI Surveillance during Pandemics: Ethical Implementation Imperatives. Hastings Cent Rep.

[52] Google Our Principles ? Google AI. Google.

[53] Osoba OA, Boudreaux B, Saunders J, Irwin JL, Mueller PA, Cherney S (2019). Algorithmic Equity: A Framework for Social Applications.

[54] Villasenor J (2020). Soft law as a complement to AI regulation. Brookings Institution.

[55] Wolff RF, Moons KGM, Riley RD, Whiting PF, Westwood M, Collins GS, Reitsma JB, Kleijnen J, Mallett S, PROBAST Group† (2019). PROBAST: A Tool to Assess the Risk of Bias and Applicability of Prediction Model Studies. Ann Intern Med.

[56] Miller K (2020). When Algorithmic Fairness Fixes Fail: The Case for Keeping Humans in the Loop: Stanford University Human-Centered Artificial Intelligence; November 2, 2020. Stanford.

[57] Bellamy RKE, Dey K, Hind M, Hoffman SC, Houde S, Kannan K, Lohia P, Martino J, Mehta S, Mojsilovic A, Nagar S, Ramamurthy KN, Richards J, Saha D, Sattigeri P, Singh M, Varshney KR, Zhang Y (2019). AI Fairness 360: An extensible toolkit for detecting and mitigating algorithmic bias. IBM J. Res. & Dev.

[58] Wawira Gichoya J, McCoy LG, Celi LA, Ghassemi M (2021). Equity in essence: a call for operationalising fairness in machine learning for healthcare. BMJ Health Care Inform.

[59] Parbhoo S, Wawira Gichoya J, Celi LA, de la Hoz MÁ, for MIT Critical Data (2022). Operationalising fairness in medical algorithms. BMJ Health Care Inform.

[60] Chin C, Robison M (2020). How AI bots and voice assistants reinforce gender bias. Brookings Institution.

