# Supplementary material for: Strategies to Improve the Impact of Artificial Intelligence on Health Equity: Scoping Review
Source: JMIR AI. 2023 Feb 7;2:e42936. doi: 10.2196/42936 (PMC11041459; doi:10.2196/42936)
Supplement: Multimedia Appendix 2 [file ai_v2i1e42936_app2.docx]

This is a Multimedia Appendix to a full manuscript submitted to the J Med Internet Res AI.

# Literature Search Documentation

We conducted systematic searches of PubMed, Web of Science, the Institute of Electrical and Electronics Engineers (IEEE) Xplore Digital Library, Proquest US Newsstream, Academic Search Complete, the ClinicalTrials.gov database, the FDA Center for Devices and Radiological Health (CDRH) document library, and Google web search.

This appendix documents the terms used in each of these searches.

**Pubmed Searches**

Notes on search fields:

[ti] = Title

[tiab] = Title and Abstract

[mh] = MeSH Terms

**PubMed Search #1**

**AI and Equity, review articles**

Search conducted on December 10, 2021

Date Range: January 1, 2014-present

English

"machine learning"[ti] OR "artificial intelligence"[ti] OR "deep learning"[ti] OR "supervised learning"[ti] OR "unsupervised learning"[ti] OR "reinforcement learning"[ti] OR "unsupervised clustering"[ti] OR "unsupervised classification"[ti] OR "supervised classification"[ti] OR "natural language processing"[ti] OR NLP[ti] OR "expert system*"[ti] OR "rules engine*"[ti] OR "fuzzy logic"[ti] OR algorithm*[ti] OR "Artificial Intelligence"[mh]
AND
health*[tiab] OR clinic*[tiab] OR patient*[tiab] OR hospital*[tiab] OR therap*[tiab] OR medic*[tiab] OR care[tiab]
AND
equit*[tiab] OR fair*[tiab] OR unfair[tiab] OR bias*[tiab] OR inequ*[tiab] OR unequ*[tiab] OR equality[tiab] OR inclusiv*[tiab] OR exclude*[tiab] OR race[tiab] OR racial[tiab] OR racism[tiab] OR gender[tiab] OR sex[tiab] OR ethnic*[tiab] OR disab*[tiab] OR dispar*[tiab] OR disproportion*[tiab] OR "social determinant*"[tiab] OR socioeconomic*[tiab] OR income[tiab] OR minorit*[tiab] OR disadvantaged[tiab] OR vulnerab*[tiab] OR marginali*[tiab] OR "Health Equity"[mh] OR "Gender Equity"[mh] OR "Healthcare Disparities"[mh] OR "Prejudice"[mh] OR "Social Determinants of Health"[mh] OR "Minority Health"[mh] OR "Racial Groups"[mh] OR "Socioeconomic Factors"[mh] OR "Race Relations"[mh] OR "Ethnicity"[mh]
AND
Review[ti]

**PubMed Search #2**

**AI and Equity in COVID-19 Response, all articles**

Search conducted on December 10, 2021

Date Range: December 31, 2019-present

English

"machine learning"[ti] OR "artificial intelligence"[ti] OR "deep learning"[ti] OR "supervised learning"[ti] OR "unsupervised learning"[ti] OR "reinforcement learning"[ti] OR "unsupervised clustering"[ti] OR "unsupervised classification"[ti] OR "supervised classification"[ti] OR "natural language processing"[ti] OR NLP[ti] OR "expert system*"[ti] OR "rules engine*"[ti] OR "fuzzy logic"[ti] OR algorithm*[ti] OR "Artificial Intelligence"[mh]
AND
Coronavirus[tiab] OR COVID*[tiab] OR "SARS-COV-2"[tiab] OR "2019-nCOV"[tiab] OR "nCOV-19"[tiab] OR "COVID-19"[mh] OR "SARS-CoV-2"[mh] OR "COVID-19 Testing"[mh]
AND
equit*[tiab] OR fair*[tiab] OR unfair[tiab] OR bias*[tiab] OR inequ*[tiab] OR unequ*[tiab] OR equality[tiab] OR inclusiv*[tiab] OR exclude*[tiab] OR race[tiab] OR racial[tiab] OR racism[tiab] OR gender[tiab] OR sex[tiab] OR ethnic*[tiab] OR disab*[tiab] OR dispar*[tiab] OR disproportion*[tiab] OR "social determinant*"[tiab] OR socioeconomic*[tiab] OR income[tiab] OR minorit*[tiab] OR disadvantaged[tiab] OR vulnerab*[tiab] OR marginali*[tiab] OR "Health Equity"[mh] OR "Gender Equity"[mh] OR "Healthcare Disparities"[mh] OR "Prejudice"[mh] OR "Social Determinants of Health"[mh] OR "Minority Health"[mh] OR "Racial Groups"[mh] OR "Socioeconomic Factors"[mh] OR "Race Relations"[mh] OR "Ethnicity"[mh]

**Pubmed Search #3**

**AI in COVID-19 Response, review articles**

Search conducted on December 10, 2021

Date Range: December 31, 2019-present

Language: English

"machine learning"[ti] OR "artificial intelligence"[ti] OR "deep learning"[ti] OR "neural net*"[ti] OR "support vector machine*"[ti] OR SVM[ti] OR "random forest*"[ti] OR "supervised learning"[ti] OR "unsupervised learning"[ti] OR "reinforcement learning"[ti] OR "unsupervised clustering"[ti] OR "unsupervised classification"[ti] OR "supervised classification"[ti] OR "natural language processing"[ti] OR NLP[ti] OR "gradient boost*"[ti] OR "ensemble model"[ti] OR "expert system*"[ti] OR "rules engine*"[ti] OR "fuzzy logic"[ti] OR algorithm*[ti] OR "Artificial Intelligence"[mh]
AND
Coronavirus[tiab] OR COVID*[tiab] OR "SARS-COV-2"[tiab] OR "2019-nCOV"[tiab] OR "nCOV-19"[tiab] OR "COVID-19"[mh] OR "SARS-CoV-2"[mh] OR "COVID-19 Testing"[mh]
AND
review[ti]

**Web of Science Searches**

*Science Citation Index Expanded (SCI-EXPANDED), Social Sciences Citation Index (SSCI), Arts & Humanities Citation Index (A&HCI), Conference Proceedings Citation Index – Science (CPCI-S), Conference Proceedings Citation Index – Social Science & Humanities (CPCI-SSH), Emerging Sources Citation Index (ESCI):*

Notes on search fields:

TI = Title

KP = Keywords Plus

AK = Author Keywords

TS = Topic

[mh] = MeSH Terms

**Web of Science Search #1**

**AI and Equity, review articles**

Search conducted on December 10, 2021
Date Range: January 1, 2014 – December 10, 2021
English

TI=("machine learning" OR "artificial intelligence" OR "deep learning" OR "supervised learning" OR "unsupervised learning" OR "reinforcement learning" OR "unsupervised clustering" OR "unsupervised classification" OR "supervised classification" OR "natural language processing" OR NLP OR "expert system*" OR "rules engine*" OR "fuzzy logic" OR algorithm*) OR KP=("machine learning" OR "artificial intelligence" OR "deep learning" OR "supervised learning" OR "unsupervised learning" OR "reinforcement learning" OR "unsupervised clustering" OR "unsupervised classification" OR "supervised classification" OR "natural language processing" OR NLP OR "expert system*" OR "rules engine*" OR "fuzzy logic" OR algorithm*) OR AK=("machine learning" OR "artificial intelligence" OR "deep learning" OR "supervised learning" OR "unsupervised learning" OR "reinforcement learning" OR "unsupervised clustering" OR "unsupervised classification" OR "supervised classification" OR "natural language processing" OR NLP OR "expert system*" OR "rules engine*" OR "fuzzy logic" OR algorithm*)
AND
TI=(health* OR clinic* OR patient* OR hospital* OR therap* OR medic* OR care) OR AB=(health* OR clinic* OR patient* OR hospital* OR therap* OR medic* OR care)
AND
TS=(equit* OR fair* OR unfair OR bias* OR inequ* OR unequ* OR equality OR inclusiv* OR exclude* OR race OR racial OR racism OR gender OR sex OR ethnic* OR disab* OR dispar* OR disproportion* OR "social determinant*" OR socioeconomic* OR income OR minorit* OR disadvantaged OR vulnerab* OR marginali* OR prejudic*)
AND
TI=(review)

**Web of Science Search #2**

**AI and Equity, highly-cited articles**^[[1]](#footnote-2)^

Search conducted on December 10, 2021
Date Range: January 1, 2014 – December 10, 2021
English

TI=("machine learning" OR "artificial intelligence" OR "deep learning" OR "supervised learning" OR "unsupervised learning" OR "reinforcement learning" OR "unsupervised clustering" OR "unsupervised classification" OR "supervised classification" OR "natural language processing" OR NLP OR "expert system*" OR "rules engine*" OR "fuzzy logic" OR algorithm*) OR AB=("machine learning" OR "artificial intelligence" OR "deep learning" OR "supervised learning" OR "unsupervised learning" OR "reinforcement learning" OR "unsupervised clustering" OR "unsupervised classification" OR "supervised classification" OR "natural language processing" OR NLP OR "expert system*" OR "rules engine*" OR "fuzzy logic" OR algorithm*)
AND
TS=(health* OR clinic* OR patient* OR hospital* OR therap* OR medic* OR care)
AND
TS=(equit* OR fair* OR unfair OR bias* OR inequ* OR unequ* OR equality OR inclusiv* OR exclude* OR race OR racial OR racism OR gender OR sex OR ethnic* OR disab* OR dispar* OR disproportion* OR "social determinant*" OR socioeconomic* OR income OR minorit* OR disadvantaged OR vulnerab* OR marginali* OR prejudic*)

**Web of Science Search #3**

**AI and Equity in COVID Response, all articles**

Search conducted on December 10, 2021
Date range: December 31, 2019 – December 10, 2021
English

TI=("machine learning" OR "artificial intelligence" OR "deep learning" OR "supervised learning" OR "unsupervised learning" OR "reinforcement learning" OR "unsupervised clustering" OR "unsupervised classification" OR "supervised classification" OR "natural language processing" OR NLP OR "expert system*" OR "rules engine*" OR "fuzzy logic" OR algorithm*) OR KP=("machine learning" OR "artificial intelligence" OR "deep learning" OR "supervised learning" OR "unsupervised learning" OR "reinforcement learning" OR "unsupervised clustering" OR "unsupervised classification" OR "supervised classification" OR "natural language processing" OR NLP OR "expert system*" OR "rules engine*" OR "fuzzy logic" OR algorithm*) OR AK=("machine learning" OR "artificial intelligence" OR "deep learning" OR "supervised learning" OR "unsupervised learning" OR "reinforcement learning" OR "unsupervised clustering" OR "unsupervised classification" OR "supervised classification" OR "natural language processing" OR NLP OR "expert system*" OR "rules engine*" OR "fuzzy logic" OR algorithm*)
AND
TS=(Coronavirus OR COVID* OR "SARS-COV-2" OR "2019-nCOV" OR "nCOV-19")
AND
TS=(equit* OR fair* OR unfair OR bias* OR inequ* OR unequ* OR equality OR inclusiv* OR exclude* OR race OR racial OR racism OR gender OR sex OR ethnic* OR disab* OR dispar* OR disproportion* OR "social determinant*" OR socioeconomic* OR income OR minorit* OR disadvantaged OR vulnerab* OR marginali* OR prejudic*)

**Web of Science Search #4**

**AI in COVID-19 Response, review articles**

Search conducted on December 10, 2021

Date range: December 31, 2019 – December 10, 2021

English

TI=("machine learning" OR "artificial intelligence" OR "deep learning" OR "neural net*" OR "support vector machine*" OR SVM OR "random forest*" OR "supervised learning" OR "unsupervised learning" OR "reinforcement learning" OR "unsupervised clustering" OR "unsupervised classification" OR "supervised classification" OR "natural language processing" OR NLP OR "gradient boost*" OR "ensemble model" OR "expert system*" OR "rules engine*" OR "fuzzy logic" OR algorithm*) OR KP=("machine learning" OR "artificial intelligence" OR "deep learning" OR "neural net*" OR "support vector machine*" OR SVM OR "random forest*" OR "supervised learning" OR "unsupervised learning" OR "reinforcement learning" OR "unsupervised clustering" OR "unsupervised classification" OR "supervised classification" OR "natural language processing" OR NLP OR "gradient boost*" OR "ensemble model" OR "expert system*" OR "rules engine*" OR "fuzzy logic" OR algorithm*) OR AK=("machine learning" OR "artificial intelligence" OR "deep learning" OR "neural net*" OR "support vector machine*" OR SVM OR "random forest*" OR "supervised learning" OR "unsupervised learning" OR "reinforcement learning" OR "unsupervised clustering" OR "unsupervised classification" OR "supervised classification" OR "natural language processing" OR NLP OR "gradient boost*" OR "ensemble model" OR "expert system*" OR "rules engine*" OR "fuzzy logic" OR algorithm*)
AND
TS=(Coronavirus OR COVID* OR "SARS-COV-2" OR "2019-nCOV" OR "nCOV-19")
AND
TI=(review)

**IEEE Xplore Searches**

**IEEE Xplore Search #1**

**AI and Equity, review articles**

Search conducted on December 13, 2021
Date Range: 2014 – 2021

(“Document Title”:”machine learning” OR “Document Title”:”artificial intelligence” OR “Document Title”:”deep learning” OR “Document Title”:”supervised learning” OR “Document Title”:”unsupervised learning” OR “Document Title”:”reinforcement learning” OR “Document Title”:”unsupervised clustering” OR “Document Title”:”unsupervised classification” OR “Document Title”:”supervised classification” OR “Document Title”:”natural language processing” OR “Document Title”:NLP OR “Document Title”:”expert system*” OR “Document Title”:”rules engine*” OR “Document Title”:”fuzzy logic” OR “Document Title”:algorithm*)

AND

(“Document Title” or “Abstract”:health* OR “Document Title” or “Abstract”:clinic* OR “Document Title” or “Abstract”:patient* OR “Document Title” or “Abstract”:hospital* OR “Document Title” or “Abstract”:therap* OR “Document Title” or “Abstract”:medic* OR “Document Title” or “Abstract”:care)

AND

(“Document Title” or “Abstract”:equity OR “Document Title” or “Abstract”:equities OR “Document Title” or “Abstract”:fair OR “Document Title” or “Abstract”:fairness OR “Document Title” or “Abstract”:unfair OR “Document Title” or “Abstract”:bias OR “Document Title” or “Abstract”:biased OR “Document Title” or “Abstract”:inequity OR “Document Title” or “Abstract”:unequity OR “Document Title” or “Abstract”:equality OR “Document Title” or “Abstract”:inclusive OR “Document Title” or “Abstract”:inclusivity OR “Document Title” or “Abstract”:exclude OR “Document Title” or “Abstract”:excluded OR “Document Title” or “Abstract”:race OR “Document Title” or “Abstract”:racial OR “Document Title” or “Abstract”:racism OR “Document Title” or “Abstract”:gender OR “Document Title” or “Abstract”:sex OR “Document Title” or “Abstract”:ethnic OR “Document Title” or “Abstract”:ethnicity OR “Document Title” or “Abstract”:disable OR “Document Title” or “Abstract”:disabled OR “Document Title” or “Abstract”:disab* OR “Document Title” or “Abstract”:disability OR “Document Title” or “Abstract”:disparity OR “Document Title” or “Abstract”:disparities OR “Document Title” or “Abstract”:disproportionate OR “Document Title” or “Abstract”:disproportional OR “Document Title” or “Abstract”:”social determinant” OR “Document Title” or “Abstract”:socioeconomic* OR “Document Title” or “Abstract”:income OR “Document Title” or “Abstract”:minority OR “Document Title” or “Abstract”:minorit* OR “Document Title” or “Abstract”:disadvantaged OR “Document Title” or “Abstract”:vulnerable OR “Document Title” or “Abstract”:vulnerabilities OR “Document Title” or “Abstract”:marginalized)

AND

(“Document Title”:Review)

**IEEE Xplore Search #2**

**AI and Equity in COVID-19 Response, all articles**

Search conducted on December 13, 2021
Date Range: 2020 – 2021

(“Document Title”:”machine learning” OR “Document Title”:”artificial intelligence” OR “Document Title”:”deep learning” OR “Document Title”:”supervised learning” OR “Document Title”:”unsupervised learning” OR “Document Title”:”reinforcement learning” OR “Document Title”:”unsupervised clustering” OR “Document Title”:”unsupervised classification” OR “Document Title”:”supervised classification” OR “Document Title”:”natural language processing” OR “Document Title”:NLP OR “Document Title”:”expert system*” OR “Document Title”:”rules engine*” OR “Document Title”:”fuzzy logic” OR “Document Title”:algorithm*)

AND

(“Document Title” or “Abstract”:Coronavirus OR “Document Title” or “Abstract”:COVID* OR “Document Title” or “Abstract”:”SARS-COV-2” OR “Document Title” or “Abstract”:”2019-nCOV” OR “Document Title” or “Abstract”:”nCOV-19”)

AND

(“Document Title” or “Abstract”:equity OR “Document Title” or “Abstract”:equities OR “Document Title” or “Abstract”:fair OR “Document Title” or “Abstract”:fairness OR “Document Title” or “Abstract”:unfair OR “Document Title” or “Abstract”:bias OR “Document Title” or “Abstract”:biased OR “Document Title” or “Abstract”:inequity OR “Document Title” or “Abstract”:unequity OR “Document Title” or “Abstract”:equality OR “Document Title” or “Abstract”:inclusive OR “Document Title” or “Abstract”:inclusivity OR “Document Title” or “Abstract”:exclude OR “Document Title” or “Abstract”:excluded OR “Document Title” or “Abstract”:race OR “Document Title” or “Abstract”:racial OR “Document Title” or “Abstract”:racism OR “Document Title” or “Abstract”:gender OR “Document Title” or “Abstract”:sex OR “Document Title” or “Abstract”:ethnic OR “Document Title” or “Abstract”:ethnicity OR “Document Title” or “Abstract”:disable OR “Document Title” or “Abstract”:disabled OR “Document Title” or “Abstract”:disab* OR “Document Title” or “Abstract”:disability OR “Document Title” or “Abstract”:disparity OR “Document Title” or “Abstract”:disparities OR “Document Title” or “Abstract”:disproportionate OR “Document Title” or “Abstract”:disproportional OR “Document Title” or “Abstract”:”social determinant” OR “Document Title” or “Abstract”:socioeconomic* OR “Document Title” or “Abstract”:income OR “Document Title” or “Abstract”:minority OR “Document Title” or “Abstract”:minorit* OR “Document Title” or “Abstract”:disadvantaged OR “Document Title” or “Abstract”:vulnerable OR “Document Title” or “Abstract”:vulnerabilities OR “Document Title” or “Abstract”:marginalized)

**IEEE Xplore Search #3**

**AI in COVID-19 Response, review articles**

Search conducted on December 13, 2021
Date Range: 2020 – 2021

(“Document Title”:”machine learning” OR “Document Title”:”artificial intelligence” OR “Document Title”:”deep learning” OR “Document Title”:”neural net*” OR “Document Title”:”support vector machine*” OR “Document Title”:SVM OR “Document Title”:”random forest*” OR “Document Title”:”supervised learning” OR “Document Title”:”unsupervised learning” OR “Document Title”:”reinforcement learning” OR “Document Title”:”unsupervised clustering” OR “Document Title”:”unsupervised classification” OR “Document Title”:”supervised classification” OR “Document Title”:”natural language processing” OR “Document Title”:NLP OR “Document Title”:”gradient boost*” OR “Document Title”:”ensemble model” OR “Document Title”:”expert system*” OR “Document Title”:”rules engine*” OR “Document Title”:”fuzzy logic” OR “Document Title”:algorithm*)

AND

(“Document Title”or “Abstract”:Coronavirus OR “Document Title” or “Abstract”:COVID* OR “Document Title” or “Abstract”:”SARS-COV-2” OR “Document Title” or “Abstract”:”2019-nCOV” OR “Document Title” or “Abstract”:”nCOV-19”)

AND

(“Document Title”:review)

**US NewsStream Searches**

Magazines, Newspapers, Reports, Blogs, Podcasts & Websites

Notes on search fields:

ti = Title

ab = Abstract

**US NewsStream Search #1**

**AI and Equity, all articles**

Search conducted on December 27, 2021
Date Range: December 31, 2019 - present

ti("machine learning" OR "artificial intelligence" OR "deep learning" OR "supervised learning" OR "unsupervised learning" OR "reinforcement learning" OR "unsupervised clustering" OR "unsupervised classification" OR "supervised classification" OR "natural language processing" OR NLP OR "expert system*" OR "rules engine*" OR "fuzzy logic" OR algorithm*) OR ab("machine learning" OR "artificial intelligence" OR "deep learning" OR "supervised learning" OR "unsupervised learning" OR "reinforcement learning" OR "unsupervised clustering" OR "unsupervised classification" OR "supervised classification" OR "natural language processing" OR NLP OR "expert system*" OR "rules engine*" OR "fuzzy logic" OR algorithm*) OR (MAINSUBJECT.EXACT("Artificial intelligence")
AND
ti(health* OR clinic* OR patient* OR hospital* OR therap* OR medic* OR care) OR ab(health* OR clinic* OR patient* OR hospital* OR therap* OR medic* OR care) MAINSUBJECT.EXACT("Health care")
AND
ti(equit* OR fair* OR unfair OR bias* OR inequ* OR unequ* OR equality OR inclusiv* OR exclude* OR race OR racial OR racism OR gender OR sex OR ethnic* OR disab* OR dispar* OR disproportion* OR "social determinant*" OR socioeconomic* OR income OR minorit* OR disadvantaged OR vulnerab* OR marginali* OR prejudic*) OR ab(equit* OR fair* OR unfair OR bias* OR inequ* OR unequ* OR equality OR inclusiv* OR exclude* OR race OR racial OR racism OR gender OR sex OR ethnic* OR disab* OR dispar* OR disproportion* OR "social determinant*" OR socioeconomic* OR income OR minorit* OR disadvantaged OR vulnerab* OR marginali* OR prejudic*) OR (MAINSUBJECT.EXACT("Distributive justice") OR MAINSUBJECT.EXACT("Discrimination") OR MAINSUBJECT.EXACT("Social exclusion") OR MAINSUBJECT.EXACT("Equality") OR MAINSUBJECT.EXACT("Inequality") OR MAINSUBJECT.EXACT("Racial justice")))

**US NewsStream Search #2**

**AI in COVID-19 Response, all articles**

Search conducted on December 27, 2021
Date Range: December 31, 2019 - present

ti("machine learning" OR "artificial intelligence" OR "deep learning" OR "neural net*" OR "support vector machine*" OR SVM OR "random forest*" OR "supervised learning" OR "unsupervised learning" OR "reinforcement learning" OR "unsupervised clustering" OR "unsupervised classification" OR "supervised classification" OR "natural language processing" OR NLP OR "gradient boost*" OR "ensemble model" OR "expert system*" OR "rules engine*" OR "fuzzy logic" OR algorithm*) OR (MAINSUBJECT.EXACT("Artificial intelligence")
AND
(ti(Coronavirus OR COVID* OR "SARS-COV-2" OR "2019-nCOV" OR "nCOV-19") or ab(Coronavirus OR COVID* OR "SARS-COV-2" OR "2019-nCOV" OR "nCOV-19"))

**Academic Search Complete Searches**

Magazines, Trade Publications, Newspapers

Notes on search fields:

ti = Title

ab = Abstract

DE = Heading or Keyword

KW = Keyword

**Academic Search Complete Search #1**

**AI and Equity, all articles**

Search conducted on December 27, 2021
Date Range: December 31, 2019 – present

TI("machine learning" OR "artificial intelligence" OR "deep learning" OR "supervised learning" OR "unsupervised learning" OR "reinforcement learning" OR "unsupervised clustering" OR "unsupervised classification" OR "supervised classification" OR "natural language processing" OR NLP OR "expert system*" OR "rules engine*" OR "fuzzy logic" OR algorithm*) OR ab("machine learning" OR "artificial intelligence" OR "deep learning" OR "supervised learning" OR "unsupervised learning" OR "reinforcement learning" OR "unsupervised clustering" OR "unsupervised classification" OR "supervised classification" OR "natural language processing" OR NLP OR "expert system*" OR "rules engine*" OR "fuzzy logic" OR algorithm*) OR DE "Artificial intelligence"
AND
TI(health* OR clinic* OR patient* OR hospital* OR therap* OR medic* OR care) OR AB(health* OR clinic* OR patient* OR hospital* OR therap* OR medic* OR care) OR KW(health* OR clinic* OR patient* OR hospital* OR therap* OR medic* OR care)
AND
TI(equit* OR fair* OR unfair OR bias* OR inequ* OR unequ* OR equality OR inclusiv* OR exclude* OR race OR racial OR racism OR gender OR sex OR ethnic* OR disab* OR dispar* OR disproportion* OR "social determinant*" OR socioeconomic* OR income OR minorit* OR disadvantaged OR vulnerab* OR marginali* OR prejudic*) OR AB(equit* OR fair* OR unfair OR bias* OR inequ* OR unequ* OR equality OR inclusiv* OR exclude* OR race OR racial OR racism OR gender OR sex OR ethnic* OR disab* OR dispar* OR disproportion* OR "social determinant*" OR socioeconomic* OR income OR minorit* OR disadvantaged OR vulnerab* OR marginali* OR prejudic*) OR (MAINSUBJECT.EXACT("Distributive justice") OR DE "HEALTH equity" OR DE "EQUITY"

**Academic Search Complete Search #2**

**AI in COVID-19 Response, all articles**

Search conducted on December 27, 2021
Date Range: December 31, 2019 - present

TI("machine learning" OR "artificial intelligence" OR "deep learning" OR "neural net*" OR "support vector machine*" OR SVM OR "random forest*" OR "supervised learning" OR "unsupervised learning" OR "reinforcement learning" OR "unsupervised clustering" OR "unsupervised classification" OR "supervised classification" OR "natural language processing" OR NLP OR "gradient boost*" OR "ensemble model" OR "expert system*" OR "rules engine*" OR "fuzzy logic" OR algorithm*) OR DE "Artificial intelligence"
AND
(ti(Coronavirus OR COVID* OR "SARS-COV-2" OR "2019-nCOV" OR "nCOV-19") or ab(Coronavirus OR COVID* OR "SARS-COV-2" OR "2019-nCOV" OR "nCOV-19")) OR DE "COVID-19"

# Stakeholder Interview Protocol

This appendix presents the interview guide used in conducting stakeholder interviews. This interview guide was sent as an email attachment to stakeholder interviewees prior to conducting the interview. This same document was also used by interviewers while they were carrying out interviews as a guide to obtaining informed concept and asking questions. It includes a study description, an informed consent protocol, and a list of potential interview questions.

The remainder of this appendix section provides the full text of the interview guide sent to stakeholder representatives prior to their participation in an interview. References to ‘you’ in this guide refer to the intended audience: a stakeholder representative participating in an upcoming interview.

## Interview Guide

The study description, informed consent protocol, and interview guide will be covered during the interview itself and will also be sent to potential interviewees in advance of any scheduled interview. Interviewers will read the following study description and informed consent protocol before beginning the interview.

### Study Description

This project is funded by the Patient Centered Outcomes Research Institute (PCORI) and is being conducted by the RAND Corporation. The goal of our study is to better understand the use of artificial intelligence in the clinical and public health response to COVID-19, including its potential impacts on health equity. We are interested in understanding the types of AI applications being used in the COVID-19 response, the functions that these applications perform, and the contexts in which they are used. We are also interested in assessing the evidence surrounding these applications’ use, in terms of both their benefits and drawbacks, particularly in regards to inter-group health disparities and equity concerns. We will seek to identify strategies to mitigate negative impacts and enhance positive impacts of AI on health equity.

To do this, we will be producing a literature and evidence review on these subjects. We are also conducting key informant interviews, in order to ensure that we take into account a range of perspectives and priorities as we determine the review scope and guiding questions. We expect the final product of our study to be a published report available on PCORI’s website.

### Informed Consent Protocol

We are conducting interviews with a wide range of people, including patients and patient advocates, clinicians, hospitals and health systems, healthcare payers and insurers, health policymakers, public health officials, industry, and researchers. We would like to interview you for this study. You have been selected because of your perspective, interest, and experience with issues relevant to this research.

**Risks:** We do not expect that you would face any risks related to your participation in this interview.

**Confidentiality:** We will keep your responses during this interview confidential. We will not be recording this interview, though we will be taking written notes. These notes will only be accessible to the study team. We will not include your name in our interview notes, and we will store all interview notes securely, and separate from the list of interviewee names and identifying information. We will be reporting themes and variation in responses evident across all of our interviews. We may refer to information or opinions you express in the interview in our report, but we will be attributing these generically to a ‘key informant interviewee,’ and will not be attributing comments to anyone by name, position, affiliation, or in any way that could be used to identify you.

**Duration:** Your participation in this interview will last about an hour.

**Participation and Withdrawal**: Participation in this interview is entirely voluntary. Deciding not to participate will have no negative consequences. If you decide to participate, you are free to end the interview at any point or decline to answer any question for any reason.

**Questions for Interviewer:** Do you have any questions about this study or about participation in this interview that you would like answered now?

**Informed Consent:** Do you consent to participate in this interview?

### Interview Guide

A list of interview questions can be found below.

While we plan to use these prompts and questions to guide our interview discussions, we will not be using these as a verbatim script for interviews. Rather, we will tailor our exact interview approach to each individual interviewee, taking into account the varying perspectives and backgrounds that different individuals bring to this topic. Some questions may not be applicable to all interviewees, in which case we will move on to another question or topic.

Throughout the interview we will encourage interviewees to expand upon their answers or raise additional topics they feel are important for discussion.

#### A. Background

Tell us a little about your background on this topic.

A1. In what ways, if any, have you been involved in or affected by the use of artificial intelligence in the clinical care or public health response to COVID-19?

A2. What experiences or other factors inform your perspective on the use of AI in health care and its potential impact on health equity?

#### B. Key Questions

Our study is focused on the use of AI in the clinical and public health response to COVID-19, including its potential impacts on health equity. This includes AI-based clinical products and tools that are used to diagnose COVID-19, evaluate patient prognosis, and assess treatment benefits and harms. This also includes AI-based applications that are used in support of public health efforts such as COVID-19 forecasting, contract tracing, resource distribution, vaccine prioritization, and combating health misinformation.

We are in the process of refining the study questions that will be used to guide our literature review. Our current list includes several key questions. We are interested in your answers to these questions as well as your thoughts on whether these are the right questions to be asking in our study.

We will first ask you to answer each of these questions directly. We know these questions cover a broad range of issues and we will not have time to address them in complete detail. So please just answer with whatever first comes to mind.

B1. Are you familiar with any AI-based applications that have been used in clinical care for COVID-19? Any that may be in use in the near-future? [If yes to either question - please describe them]

B2. Are you familiar with any AI-based applications that have been used in the public health response to COVID-19? Any that may be in use in the near-future? [If yes to either question – please describe them]

B3. Are you aware of any evidence that is available on the potential benefits and drawbacks of these applications? [If yes – please elaborate]

B4. What potential negative impacts of the use of AI on health equity are you aware of or concerned about? Are there any potential positive impacts of AI on health equity that you are aware of?

B5. Are there any strategies you are aware of that seek to mitigate negative impacts or enhance positive impacts of AI on health equity?

#### C. Research Priorities

We appreciate your answers to those questions, as this is important information for our study. We’d like to continue to talk about these same topics, though taking a step back to think about them a bit differently, since we’d like to get your opinion on which aspects of these topics you think we should focus our research on.

As I mentioned earlier, PCORI has asked us to produce a literature review on the use of AI in the COVID-19 response and its impact on health equity.

This literature review has two goals. First, it is intended to help them, and others involved in health care such as yourself, to understand the current state of the field. Secondly, this report should identify strategies that can help mitigate negative impacts and enhance positive impacts of AI on health equity.

We are just starting this project, so are very interested in getting your perspective as we determine our study scope and key research questions to help us address these goals.

C1. What questions about AI in the COVID-19 response, and the evidence surrounding its use, should we seek to answer in our literature review?

C2. Are there specific types of AI applications that we should make sure to include in our review?

C3. What types of potential benefits, harms, and other impacts should we make sure to examine in our review?

C4. What questions about how the use of AI in health care affects health equity should we seek to answer in our literature review?

C5. What types of health equity impacts should we make sure to examine? Are there impacts on particular groups that we should make sure to look at?

C6. Are there any particular types of documents or data sources that you think would be especially relevant and useful for us to examine in our study?

1. For information on the Web of Science selection of highly cited papers, see: Web of Science Core Collection Help: Citation Products. https://images.webofknowledge.com/WOKRS533JR18/help/WOS/hs_citation_applications.html. Published 2020. Accessed December 30, 2021. [↑](#footnote-ref-2)
