# Supplementary material for: Strategies to Improve the Impact of Artificial Intelligence on Health Equity: Scoping Review
Source: JMIR AI. 2023 Feb 7;2:e42936. doi: 10.2196/42936 (PMC11041459; doi:10.2196/42936)
Supplement: Multimedia Appendix 3 [file ai_v2i1e42936_app3.docx]

Multimedia Appendix 3

Notes from Stakeholder Interviews

A summary of interviewee responses to interview prompts is as follows:

- **Overall research topic:**  None of the interviewees suggested changes to the study topics proposed for our review.
- **Datasets and health equity**: Six interviewees were interested in understanding how often subpopulation characteristics are reported for the datasets used to train, validate, or evaluate AI applications, as well as how these subpopulations were defined. Four interviewees mentioned the challenges involved in collecting data related to health disparities, given that disparities are often driven by social determinants of health that are difficult to measure, and that categories used to characterize people may be contested or poorly defined. Three interviewees mentioned that there is a tension between ensuring that data used in AI applications are inclusive of underrepresented groups on the one hand (which may require asking for and storing sensitive information such as on patient-reported race and ethnicity) and ensuring that patient privacy and trust are maintained on the other.
- **Variables relevant to health equity**: Four interviewees mentioned the use of geography as an important and readily available determinant of health, though some cautioned that it can be an imperfect proxy or can render disadvantaged groups invisible if they are dispersed over a wide area rather than geographically concentrated in a particular zip code or neighborhood. Two interviewees mentioned that AI algorithms can identify (and thus take into account) race, ethnicity, or other sensitive personal attributes even from seemingly unrelated information, such as medical images. When asked about the personal characteristics most relevant to understanding health equity, interviewees mentioned geography/location, education, housing status, immigration status, employment status, insurance status, incarceration status, access to transport, and family structure, in addition to characteristics such as race, ethnicity, gender, socioeconomic status, and disability.
- **Evaluating equity-related outcomes**: Five interviewees were interested in the topic of AI model acceptability to different types of patients, including whether patients are comfortable sharing potentially sensitive information that may be needed as data inputs. Four interviewees mentioned the importance of AI impacts on cost of care, access to care, or quality of care received, as important from an equity perspective, in addition to impacts on measurable health outcomes.
- **Availability of equity-related information on AI algorithms**: Four interviewees mentioned that AI may be used internally by an organization (such as a health system, government agency, insurance company, or technology company), but little information may be publicly available on these algorithms. This was variously attributed to concerns over publishing proprietary information, lags in academic publishing timelines, and, in one case, the fear that publishing information on algorithm use in health care could lead to bad publicity if that algorithm was determined to be biased.
- **Recommended documents and data sources**: Interviewees recommended several documents to screen for inclusion in our review, including peer-reviewed papers and grey literature documents. Interviewees also recommended more general data sources to examine in our review, including paper authors, software developers, and types of articles such as conference proceedings as well as viewpoints published in academic journals and commentaries in the news media and grey literature.
