# Supplementary material for: Strategies to Improve the Impact of Artificial Intelligence on Health Equity: Scoping Review
Source: JMIR AI. 2023 Feb 7;2:e42936. doi: 10.2196/42936 (PMC11041459; doi:10.2196/42936)
Supplement: Multimedia Appendix 4 [file ai_v2i1e42936_app4.docx]

| **Document** | **Document Type** | **Issue** | **Strategy** |
| --- | --- | --- | --- |
| Chin C, Robison M. How AI bots and voice assistants reinforce gender bias. 2020. 2020-11-23. https://www.brookings.edu/research/how-ai-bots-and-voice-assistants-reinforce-gender-bias/ | Grey Literature | Biased or Non-Representative Developers | Foster Diversity |
| D. Leslie; A. Mazumder; A. Peppin; M. K. Wolters; A. Hagerty (2021). "Does AI" stand for augmenting inequality in the era of covid-19 healthcare?"." Bmj 372():n304. 10.1136/bmj.n304 | Peer-Reviewed Literature | Biased or Non-Representative Developers | Foster Diversity |
| I. Vourganas; V. Stankovic; L. Stankovic (2020). "Individualised Responsible Artificial Intelligence for Home-Based Rehabilitation." Sensors (Basel) 21(1):. 10.3390/s21010002 | Peer-Reviewed Literature | Biased or Non-Representative Developers | Foster Diversity |
| Kenneth Holstein, Jennifer Wortman Vaughan, Hal Daumé, Miro Dudik, and Hanna Wallach. 2019. Improving Fairness in Machine Learning Systems: What Do Industry Practitioners Need? In Proceedings of the 2019 CHI Conference on Human Factors in Computing Systems (CHI '19). Association for Computing Machinery, New York, NY, USA, Paper 600, 1–16. DOI:https://doi.org/10.1145/3290605.3300830 | Grey Literature (Conference Proceedings) | Biased or Non-Representative Developers | Foster Diversity |
| O'Brien N, et al (2022). Addressing racial and ethnic inequities in data-driven health technologies. Imperial College. | Grey Literature | Biased or Non-Representative Developers | Foster Diversity |
| The Alan Turing Institute. Data science and AI in the age of COVID-19: Reflections on the response of the UK’s data science and AI community to the COVID-19 pandemic. 2021. | Grey Literature | Biased or Non-Representative Developers | Foster Diversity |
| Zdawxzyk C., Vallee A. B. (2021). Gender bias in AI: what the United States, Canada, and Western European nations can learn from other countries. OECD.AI | Grey Literature | Biased or Non-Representative Developers | Foster Diversity |
| Chin C, Robison M. How AI bots and voice assistants reinforce gender bias. 2020. 2020-11-23. https://www.brookings.edu/research/how-ai-bots-and-voice-assistants-reinforce-gender-bias/ | Grey Literature | Biased or Non-Representative Developers | Train Developers and Users |
| H. Ledford (2019). "Millions of black people affected by racial bias in health-care algorithms." Nature 574, 608-609. 10.1038/d41586-019-03228-6 | Grey Literature (News Media) | Biased or Non-Representative Developers | Train Developers and Users |
| D. Leslie; A. Mazumder; A. Peppin; M. K. Wolters; A. Hagerty (2021). "Does AI" stand for augmenting inequality in the era of covid-19 healthcare?"." Bmj 372():n304. 10.1136/bmj.n304 | Peer-Reviewed Literature | Biased or Non-Representative Developers | Engage the Broader Community |
| Rajkomar A, Hardt M, Howell MD, Corrado G, Chin MH. Ensuring Fairness in Machine Learning to Advance Health Equity. Ann Intern Med. 2018 Dec 18;169(12):866-872. doi: 10.7326/M18-1990. Epub 2018 Dec 4. PMID: 30508424; PMCID: PMC6594166. | Peer-Reviewed Literature | Biased or Non-Representative Developers | Engage the Broader Community |
| Zimmer M, Franco Z, Madiraju P, Echeveste C, Heindel K, Ogle J. Public Opinion Research on Artificial Intelligence in Public Health Responses: Results of Focus Groups with Four Communities. Washington, DC: AAAS Center for Public Engagement with Science and Technology;2021. | Grey Literature | Biased or Non-Representative Developers | Engage the Broader Community |
| Kenneth Holstein, Jennifer Wortman Vaughan, Hal Daumé, Miro Dudik, and Hanna Wallach. 2019. Improving Fairness in Machine Learning Systems: What Do Industry Practitioners Need? In Proceedings of the 2019 CHI Conference on Human Factors in Computing Systems (CHI '19). Association for Computing Machinery, New York, NY, USA, Paper 600, 1–16. DOI:https://doi.org/10.1145/3290605.3300830 | Grey Literature (Conference Proceedings) | Biased or Non-Representative Developers | Increase Model Reporting and Transparency |
| M. Kiener (2020). "Artificial intelligence in medicine and the disclosure of risks." AI Soc ():44205. 10.1007/s00146-020-01085-w | Peer-Reviewed Literature | Diminished Accountability | Train Developers and Users |
| Osoba, Osonde A., Benjamin Boudreaux, Jessica Saunders, J. Luke Irwin, Pam A. Mueller, and Samantha Cherney, Algorithmic Equity: A Framework for Social Applications. Santa Monica, CA: RAND Corporation, 2019. | Grey Literature | Diminished Accountability | Evaluate Disparities in Model Performance |
| Rose S, Bergquist SL, Layton TJ. Computational health economics for identification of unprofitable health care enrollees. Biostatistics. Oct 1 2017;18(4):682-694. doi:10.1093/biostatistics/kxx012 | Peer-Reviewed Literature | Enabling Discrimination | Improve Governance |
| BBC News. IBM abandons 'biased' facial recognition tech. June 9. https://www.bbc.com/news/technology-52978191 | Grey Literature (News Media) | Enabling Discrimination | Avoid or Reduce Use of AI |
| Johnson S. A.I. Is Mastering Language. Should We Trust What It Says? New York Times Magazine; April 15, 2022. | Grey Literature (News Media) | Enabling Discrimination | Avoid or Reduce Use of AI |
| Ross C. As the FDA clears a flood of AI tools, missing data raise troubling questions on safety and fairness. Stat; 2021-02-03 February 3, 2021. | Grey Literature (News Media) | Limited or Poor Info on Population Characteristics | Improve Governance |
| Morey BN, Chang RC, Thomas KB, et al. No Equity without Data Equity: Data Reporting Gaps for Native Hawaiians and Pacific Islanders as Structural Racism. Journal of Health Politics, Policy and Law. 2022;47(2):159-200. doi:10.1215/03616878-9517177 | Peer-Reviewed Literature | Limited or Poor Info on Population Characteristics | Include Sensitive Variables to Correct for Bias |
| O'Neill J, Tabish H, Welch V, et al. Applying an equity lens to interventions: using PROGRESS ensures consideration of socially stratifying factors to illuminate inequities in health. J Clin Epidemiol. 2014/01/01/ 2014;67(1):56-64. doi:https://doi.org/10.1016/j.jclinepi.2013.08.005 | Peer-Reviewed Literature | Limited or Poor Info on Population Characteristics | Increase Model Reporting and Transparency |
| Ross C. As the FDA clears a flood of AI tools, missing data raise troubling questions on safety and fairness. Stat; 2021-02-03 February 3, 2021. | Grey Literature (News Media) | Limited or Poor Info on Population Characteristics | Increase Model Reporting and Transparency |
| A. Deonarine; G. Lyons; C. Lakhani; W. De Brouwer (2021). "Identifying Communities at Risk for COVID-19-Related Burden Across 500 US Cities and Within New York City: Unsupervised Learning of the Coprevalence of Health Indicators." JMIR Public Health Surveill 7(8):e26604. 10.2196/26604 | Peer-Reviewed Literature | Limited or Poor Info on Population Characteristics | Improve Diversity, Quality, or Quantity of Data |
| A. Kundu; M. Chaiton; R. Billington; D. Grace; R. Fu; C. Logie; B. Baskerville; C. Yager; N. Mitsakakis; R. Schwartz (2021). "Machine Learning Applications in Mental Health and Substance Use Research Among the LGBTQ2S+ Population: Scoping Review." JMIR Med Inform 9(11):e28962. 10.2196/28962 | Peer-Reviewed Literature | Limited or Poor Info on Population Characteristics | Improve Diversity, Quality, or Quantity of Data |
| Mhasawade V, Zhao Y, Chunara R. Machine learning and algorithmic fairness in public and population health. Nat Mach Intell. 2021/08/01 2021;3(8):659-666. doi:10.1038/s42256-021-00373-4 | Peer-Reviewed Literature | Limited or Poor Info on Population Characteristics | Improve Diversity, Quality, or Quantity of Data |
| Obermeyer Z, Nissan R, Stern M, Eaneff S, Bembeneck EJ, Mullainathan S. Algorithmic Bias Playbook. The Center for Applied Artificial Intelligence, Chicago Booth;June 2021. | Grey Literature | Limited or Poor Info on Population Characteristics | Improve Diversity, Quality, or Quantity of Data |
| Stevens LA, Claybon MA, Schmid CH, et al. Evaluation of the Chronic Kidney Disease Epidemiology Collaboration equation for estimating the glomerular filtration rate in multiple ethnicities. Kidney international. 2011;79(5):555-562. | Peer-Reviewed Literature | Limited or Poor Info on Population Characteristics | Improve Diversity, Quality, or Quantity of Data |
| The Alan Turing Institute. Data science and AI in the age of COVID-19: Reflections on the response of the UK’s data science and AI community to the COVID-19 pandemic. 2021. | Grey Literature | Limited or Poor Info on Population Characteristics | Improve Diversity, Quality, or Quantity of Data |
| UnitedHealthcare and the AMA Collaborate to Understand and Address Social Barriers Preventing People’s Access to Better Health. April 2, 2019. https://www.unitedhealthgroup.com/newsroom/2019/2019-04-02-uhc-ama-social-barriers.html | Grey Literature (Press Release) | Limited or Poor Info on Population Characteristics | Improve Diversity, Quality, or Quantity of Data |
| Margaret Mitchell, et al. Model cards for model reporting. Proceedings of the Conference on Fairness, Accountability, and Transparency, Association for Computing Machinery, ACM Digital Library (2019), pp. 220-229, 10.1145/3287560.3287596 | Grey Literature (Conference Proceedings) | Limited or Poor Info on Population Characteristics | Use Equity-Focused Checklists, Guidelines, and Similar Tools |
| O'Neill J, Tabish H, Welch V, et al. Applying an equity lens to interventions: using PROGRESS ensures consideration of socially stratifying factors to illuminate inequities in health. J Clin Epidemiol. 2014/01/01/ 2014;67(1):56-64. doi:https://doi.org/10.1016/j.jclinepi.2013.08.005 | Peer-Reviewed Literature | Limited or Poor Info on Population Characteristics | Use Equity-Focused Checklists, Guidelines, and Similar Tools |
| Q. Pham; A. Gamble; J. Hearn; J. A. Cafazzo (2021). "The Need for Ethnoracial Equity in Artificial Intelligence for Diabetes Management: Review and Recommendations." J Med Internet Res 23(2):e22320. 10.2196/22320 | Peer-Reviewed Literature | Limited or Poor Info on Population Characteristics | Use Equity-Focused Checklists, Guidelines, and Similar Tools |
| M. Kiener (2020). "Artificial intelligence in medicine and the disclosure of risks." AI Soc ():44205. 10.1007/s00146-020-01085-w | Peer-Reviewed Literature | Unrepresentative Data or Small Sample Sizes | Train Developers and Users |
| J. Morley; C. C. V. Machado; C. Burr; J. Cowls; I. Joshi; M. Taddeo; L. Floridi (2020). "The ethics of AI in health care: A mapping review." Soc Sci Med 260():113172. 10.1016/j.socscimed.2020.113172 | Peer-Reviewed Literature | Unrepresentative Data or Small Sample Sizes | Engage the Broader Community |
| Reddy; S. Allan; S. Coghlan; P. Cooper (2020). "A governance model for the application of AI in health care." Journal of the American Medical Informatics Association 27(3):491-497. 10.1093/jamia/ocz192 | Peer-Reviewed Literature | Unrepresentative Data or Small Sample Sizes | Engage the Broader Community |
| U.S. Food and Drug Administration, Health Canada, United Kingdom Medicines and Healthcare products Regulatory Agency. Good Machine Learning Practice for Medical Device Development: Guiding Principles. 2021. | Grey Literature | Unrepresentative Data or Small Sample Sizes | Improve Governance |
| N. Peiffer-Smadja; T. M. Rawson; R. Ahmad; A. Buchard; P. Georgiou; F. X. Lescure; G. Birgand; A. H. Holmes (2020). "Machine learning for clinical decision support in infectious diseases: a narrative review of current applications." Clin Microbiol Infect 26(5):584-595. 10.1016/j.cmi.2019.09.009 | Peer-Reviewed Literature | Unrepresentative Data or Small Sample Sizes | Include Sensitive Variables to Correct for Bias |
| Vokinger KN, Feuerriegel S, Kesselheim AS. Mitigating bias in machine learning for medicine. Communications Medicine. 2021/08/23 2021;1(1):25. doi:10.1038/s43856-021-00028-w | Peer-Reviewed Literature | Unrepresentative Data or Small Sample Sizes | Enforce Fairness Goals |
| N. Peiffer-Smadja; T. M. Rawson; R. Ahmad; A. Buchard; P. Georgiou; F. X. Lescure; G. Birgand; A. H. Holmes (2020). "Machine learning for clinical decision support in infectious diseases: a narrative review of current applications." Clin Microbiol Infect 26(5):584-595. 10.1016/j.cmi.2019.09.009 | Peer-Reviewed Literature | Unrepresentative Data or Small Sample Sizes | Evaluate Disparities in Model Performance |
| Rodolfa KT, Salomon E, Haynes L, Mendieta IH, Larson J, Ghani R. Case study: predictive fairness to reduce misdemeanor recidivism through social service interventions. presented at: Proceedings of the 2020 Conference on Fairness, Accountability, and Transparency; 2020; Barcelona, Spain. https://doi.org/10.1145/3351095.3372863 | Grey Literature (Conference Proceedings) | Unrepresentative Data or Small Sample Sizes | Evaluate Disparities in Model Performance |
| T. Grote; P. Berens (2020). "On the ethics of algorithmic decision-making in healthcare." Journal of Medical Ethics 46(3):205-211. 10.1136/medethics-2019-105586 | Peer-Reviewed Literature | Unrepresentative Data or Small Sample Sizes | Evaluate Disparities in Model Performance |
| S. H. Park; Y. H. Kim; J. Y. Lee; S. Yoo; C. J. Kim (2019). "Ethical challenges regarding artificial intelligence in medicine from the perspective of scientific editing and peer review." Science Editing 6(2):91-98. 10.6087/kcse.164 | Peer-Reviewed Literature | Unrepresentative Data or Small Sample Sizes | Increase Model Reporting and Transparency |
| T. Hernandez-Boussard; S. Bozkurt; J. P. A. Ioannidis; N. H. Shah (2020). "MINIMAR (MINimum Information for Medical Al Reporting): Developing reporting standards for artificial intelligence in health care." Journal of the American Medical Informatics Association 27(12):2011-2015. 10.1093/jamia/ocaa088 | Peer-Reviewed Literature | Unrepresentative Data or Small Sample Sizes | Increase Model Reporting and Transparency |
| Vokinger KN, Feuerriegel S, Kesselheim AS. Mitigating bias in machine learning for medicine. Communications Medicine. 2021/08/23 2021;1(1):25. doi:10.1038/s43856-021-00028-w | Peer-Reviewed Literature | Unrepresentative Data or Small Sample Sizes | Increase Model Reporting and Transparency |
| Wynants L, Smits L J M, Van Calster B. Demystifying AI in healthcare BMJ 2020; 370 :m3505 doi:10.1136/bmj.m3505 | Peer-Reviewed Literature | Unrepresentative Data or Small Sample Sizes | Increase Model Reporting and Transparency |
| T. Grote; P. Berens (2020). "On the ethics of algorithmic decision-making in healthcare." Journal of Medical Ethics 46(3):205-211. 10.1136/medethics-2019-105586 | Peer-Reviewed Literature | Unrepresentative Data or Small Sample Sizes | Avoid or Reduce Use of AI |
| A. Arora (2020). "Conceptualising Artificial Intelligence as a Digital Healthcare Innovation: An Introductory Review." Med Devices (Auckl) 13():223-230. 10.2147/mder.S262590 | Peer-Reviewed Literature | Unrepresentative Data or Small Sample Sizes | Improve Diversity, Quality, or Quantity of Data |
| A. Chouldechova, A. Roth (2018). The frontiers of fairness in machine learning. Computing research repository (CoRR) Oct. abs/1810.08810. Available at: https://arxiv.org/abs/1810.08810 | Grey Literature (Preprint) | Unrepresentative Data or Small Sample Sizes | Improve Diversity, Quality, or Quantity of Data |
| E. Röösli; B. Rice; T. Hernandez-Boussard (2021). "Bias at warp speed: how AI may contribute to the disparities gap in the time of COVID-19." J Am Med Inform Assoc 28(1):190-192. 10.1093/jamia/ocaa210 | Peer-Reviewed Literature | Unrepresentative Data or Small Sample Sizes | Improve Diversity, Quality, or Quantity of Data |
| Google. Case Study: Responsible AI: The Fitbit Blood Oxygen Saturation (SpO2) Tracking Feature. https://ai.google/static/documents/case-study-blood-oxygen-saturation-tracking.pdf | Grey Literature | Unrepresentative Data or Small Sample Sizes | Improve Diversity, Quality, or Quantity of Data |
| H. R. Liu; L. Li; I. M. Wormstone; C. Y. Qiao; C. Zhang; P. Liu; S. N. Li; H. Z. Wang; D. P. Mou; R. Q. Pang; D. Y. Yang; L. M. Zangwill; S. Moghimi; H. Y. Hou; C. Bowd; L. Jiang; Y. H. Chen; M. Hu; Y. L. Xu; H. Kang; X. Ji; R. Chang; C. Tham; C. Cheung; D. S. W. Ting; T. Y. Wong; Z. L. Wang; R. N. Weinreb; M. I. Xu; N. L. Wang (2019). "Development and Validation of a Deep Learning System to Detect Glaucomatous Optic Neuropathy Using Fundus Photographs." Jama Ophthalmology 137(12):1353-1360. 10.1001/jamaophthalmol.2019.3501 | Peer-Reviewed Literature | Unrepresentative Data or Small Sample Sizes | Improve Diversity, Quality, or Quantity of Data |
| I. G. Asiimwe; E. J. Zhang; R. Osanlou; A. L. Jorgensen; M. Pirmohamed (2021). "Warfarin dosing algorithms: A systematic review." Br J Clin Pharmacol 87(4):1717-1729. 10.1111/bcp.14608 | Peer-Reviewed Literature | Unrepresentative Data or Small Sample Sizes | Improve Diversity, Quality, or Quantity of Data |
| I. Vourganas; V. Stankovic; L. Stankovic (2020). "Individualised Responsible Artificial Intelligence for Home-Based Rehabilitation." Sensors (Basel) 21(1):. 10.3390/s21010002 | Peer-Reviewed Literature | Unrepresentative Data or Small Sample Sizes | Improve Diversity, Quality, or Quantity of Data |
| IEEE, Ethically Aligned Design: A Vision for Prioriyizing Human Well-being with Autonomous and Intelligence Systems, Version 2.The IEEE Global Initiative on Ethics of Autonomous and Intelligent Systems. Ethically Aligned Design: A Vision for Prioritizing Human Well-being with Autonomous and Intelligent Systems, First Edition. IEEE, 2019. https://standards.ieee.org/content/ieee-standards/en/industry-connections/ec/ autonomous-systems.html | Grey Literature (Conference Proceedings) | Unrepresentative Data or Small Sample Sizes | Improve Diversity, Quality, or Quantity of Data |
| J. C. Sipior (2020). "Considerations for development and use of AI in response to COVID-19." International Journal of Information Management 55():6. 10.1016/j.ijinfomgt.2020.102170 | Peer-Reviewed Literature | Unrepresentative Data or Small Sample Sizes | Improve Diversity, Quality, or Quantity of Data |
| J. Gallifant; J. Zhang; M. Del Pilar Arias Lopez; T. Zhu; L. Camporota; L. A. Celi; F. Formenti (2021). "Artificial intelligence for mechanical ventilation: systematic review of design, reporting standards, and bias." Br J Anaesth ():. 10.1016/j.bja.2021.09.025 | Peer-Reviewed Literature | Unrepresentative Data or Small Sample Sizes | Improve Diversity, Quality, or Quantity of Data |
| K. Ferryman, M. Pitcan (2018). Fairness in Precision Medicine. https://datasociety.net/library/fairness-in-precision-medicine/ | Grey Literature | Unrepresentative Data or Small Sample Sizes | Improve Diversity, Quality, or Quantity of Data |
| K. P. Seastedt; D. Moukheiber; S. A. Mahindre; C. Thammineni; D. T. Rosen; A. A. Watkins; D. A. Hashimoto; C. D. Hoang; J. Kpodonu; L. A. Celi (2021). "A scoping review of artificial intelligence applications in thoracic surgery." Eur J Cardiothorac Surg ():. 10.1093/ejcts/ezab422 | Peer-Reviewed Literature | Unrepresentative Data or Small Sample Sizes | Improve Diversity, Quality, or Quantity of Data |
| K. Wilkinson; L. Sheets; D. Fitch; L. Popejoy (2021). "Systematic review of approaches to use of neighborhood-level risk factors with clinical data to predict clinical risk and recommend interventions." J Biomed Inform 116():103713. 10.1016/j.jbi.2021.103713 | Peer-Reviewed Literature | Unrepresentative Data or Small Sample Sizes | Improve Diversity, Quality, or Quantity of Data |
| M. A. Gianfrancesco; S. Tamang; J. Yazdany; G. Schmajuk (2018). "Potential Biases in Machine Learning Algorithms Using Electronic Health Record Data." Jama Internal Medicine 178(11):1544-1547. 10.1001/jamainternmed.2018.3763 | Peer-Reviewed Literature | Unrepresentative Data or Small Sample Sizes | Improve Diversity, Quality, or Quantity of Data |
| Margaret Mitchell, et al. Model cards for model reporting. Proceedings of the Conference on Fairness, Accountability, and Transparency, Association for Computing Machinery, ACM Digital Library (2019), pp. 220-229, 10.1145/3287560.3287596 | Grey Literature (Conference Proceedings) | Unrepresentative Data or Small Sample Sizes | Improve Diversity, Quality, or Quantity of Data |
| N. Peiffer-Smadja; T. M. Rawson; R. Ahmad; A. Buchard; P. Georgiou; F. X. Lescure; G. Birgand; A. H. Holmes (2020). "Machine learning for clinical decision support in infectious diseases: a narrative review of current applications." Clin Microbiol Infect 26(5):584-595. 10.1016/j.cmi.2019.09.009 | Peer-Reviewed Literature | Unrepresentative Data or Small Sample Sizes | Improve Diversity, Quality, or Quantity of Data |
| Obermeyer Z, Nissan R, Stern M, Eaneff S, Bembeneck EJ, Mullainathan S. Algorithmic Bias Playbook. The Center for Applied Artificial Intelligence, Chicago Booth;June 2021. | Grey Literature | Unrepresentative Data or Small Sample Sizes | Improve Diversity, Quality, or Quantity of Data |
| O'Brien N, et al (2022). Addressing racial and ethnic inequities in data-driven health technologies. Imperial College. | Grey Literature | Unrepresentative Data or Small Sample Sizes | Improve Diversity, Quality, or Quantity of Data |
| Paul, C. Jolley & A. Anthony. Reflecting the Past, Shaping the Future: Making AI Work for International Development. Retrieved from USAID: https://www.usaid.gov/sites/default/files/documents/15396/AI-ML-in-Development.pdf, 2018. | Grey Literature | Unrepresentative Data or Small Sample Sizes | Improve Diversity, Quality, or Quantity of Data |
| Rajkomar A, Hardt M, Howell MD, Corrado G, Chin MH. Ensuring Fairness in Machine Learning to Advance Health Equity. Ann Intern Med. 2018 Dec 18;169(12):866-872. doi: 10.7326/M18-1990. Epub 2018 Dec 4. PMID: 30508424; PMCID: PMC6594166. | Peer-Reviewed Literature | Unrepresentative Data or Small Sample Sizes | Improve Diversity, Quality, or Quantity of Data |
| Rodolfa KT, Saleiro P, Ghani R. Bias and Fairness (Chapter 11). Big Data and Social Science. 2019. | Grey Literature (Textbook) | Unrepresentative Data or Small Sample Sizes | Improve Diversity, Quality, or Quantity of Data |
| S. M. Khan; X. X. Liu; S. Nath; E. Korot; L. Faes; S. K. Wagner; P. A. Keane; N. J. Sebire; M. J. Burton; A. K. Denniston (2021). "A global review of publicly available datasets for ophthalmological imaging: barriers to access, usability, and generalisability." Lancet Digital Health 3(1):E51-E66. 10.1016/s2589-7500(20)30240-5 | Peer-Reviewed Literature | Unrepresentative Data or Small Sample Sizes | Improve Diversity, Quality, or Quantity of Data |
| Testing the Accuracy of a Digital Test to Diagnose Covid-19. ClinicalTrials.gov. Accessed March 1, 2022, https://clinicaltrials.gov/ct2/show/NCT04407585 | Grey Literature (Clinical Trial Record) | Unrepresentative Data or Small Sample Sizes | Improve Diversity, Quality, or Quantity of Data |
| The “All of Us” Research Program. N Engl J Med. 2019;381(7):668-676. | Peer-Reviewed Literature | Unrepresentative Data or Small Sample Sizes | Improve Diversity, Quality, or Quantity of Data |
| Thinking on its own: AI in the NHS (2018) | Grey Literature | Unrepresentative Data or Small Sample Sizes | Improve Diversity, Quality, or Quantity of Data |
| Vokinger KN, Feuerriegel S, Kesselheim AS. Mitigating bias in machine learning for medicine. Communications Medicine. 2021/08/23 2021;1(1):25. doi:10.1038/s43856-021-00028-w | Peer-Reviewed Literature | Unrepresentative Data or Small Sample Sizes | Improve Diversity, Quality, or Quantity of Data |
| Wynants L, Smits L J M, Van Calster B. Demystifying AI in healthcare BMJ 2020; 370 :m3505 doi:10.1136/bmj.m3505 | Peer-Reviewed Literature | Unrepresentative Data or Small Sample Sizes | Improve Diversity, Quality, or Quantity of Data |
| Simonite T. When It Comes to Health Care, AI Has a Long Way to Go. Wired. January 16, 2022. https://www.wired.com/story/health-care-ai-long-way-to-go/ | Grey Literature (News Media) | Unrepresentative Data or Small Sample Sizes | Improve Diversity, Quality, or Quantity of Data |
| Bozkurt S, Cahan EM, Seneviratne MG, Sun R, Lossio-Ventura JA, Ioannidis JPA, Hernandez-Boussard T. Reporting of demographic data and representativeness in machine learning models using electronic health records. J Am Med Inform Assoc. 2020 Dec 9;27(12):1878-1884. doi: 10.1093/jamia/ocaa164. PMID: 32935131; PMCID: PMC7727384. | Peer-Reviewed Literature | Unrepresentative Data or Small Sample Sizes | Use Equity-Focused Checklists, Guidelines, and Similar Tools |
| D. Leslie; A. Mazumder; A. Peppin; M. K. Wolters; A. Hagerty (2021). "Does AI" stand for augmenting inequality in the era of covid-19 healthcare?"." Bmj 372():n304. 10.1136/bmj.n304 | Peer-Reviewed Literature | Unrepresentative Data or Small Sample Sizes | Use Equity-Focused Checklists, Guidelines, and Similar Tools |
| Obermeyer Z, Nissan R, Stern M, Eaneff S, Bembeneck EJ, Mullainathan S. Algorithmic Bias Playbook. The Center for Applied Artificial Intelligence, Chicago Booth;June 2021. | Grey Literature | Unrepresentative Data or Small Sample Sizes | Use Equity-Focused Checklists, Guidelines, and Similar Tools |
| Osoba, Osonde A., Benjamin Boudreaux, Jessica Saunders, J. Luke Irwin, Pam A. Mueller, and Samantha Cherney, Algorithmic Equity: A Framework for Social Applications. Santa Monica, CA: RAND Corporation, 2019. | Grey Literature | Unrepresentative Data or Small Sample Sizes | Use Equity-Focused Checklists, Guidelines, and Similar Tools |
| M. Kiener (2020). "Artificial intelligence in medicine and the disclosure of risks." AI Soc ():44205. 10.1007/s00146-020-01085-w | Peer-Reviewed Literature | Bias Ingrained in Data | Train Developers and Users |
| C. J. Kelly; A. Karthikesalingam; M. Suleyman; G. Corrado; D. King (2019). "Key challenges for delivering clinical impact with artificial intelligence." Bmc Medicine 17(1):9. 10.1186/s12916-019-1426-2 | Peer-Reviewed Literature | Bias Ingrained in Data | Engage the Broader Community |
| Cathy O’Neil. Weapons of math destruction: how big data increases inequality and threatens democracy. Crown Publishing Group, USA (2016) | Peer-Reviewed Literature | Bias Ingrained in Data | Exclude Sensitive Variables to Correct for Bias |
| Darshali A. Vyas, et al. Hidden in Plain Sight — Reconsidering the Use of Race Correction in Clinical Algorithms. N Engl J Med, 0 (June (0)) (2020), 10.1056/NEJMms2004740 | Peer-Reviewed Literature | Bias Ingrained in Data | Exclude Sensitive Variables to Correct for Bias |
| A. Chouldechova, A. Roth (2018). The frontiers of fairness in machine learning. Computing research repository (CoRR) Oct. abs/1810.08810. Available at: https://arxiv.org/abs/1810.08810 | Grey Literature (Preprint) | Bias Ingrained in Data | Enforce Fairness Goals |
| Chen IY, Pierson E, Rose S, Joshi S, Ferryman K, Ghassemi M. Ethical Machine Learning in Healthcare. Annual Review of Biomedical Data Science. 2021;4(1):123-144. doi:10.1146/annurev-biodatasci-092820-114757 | Peer-Reviewed Literature | Bias Ingrained in Data | Enforce Fairness Goals |
| O'Brien N, et al (2022). Addressing racial and ethnic inequities in data-driven health technologies. Imperial College. | Grey Literature | Bias Ingrained in Data | Enforce Fairness Goals |
| Rodolfa KT, Saleiro P, Ghani R. Bias and Fairness (Chapter 11). Big Data and Social Science. 2019. | Grey Literature (Textbook) | Bias Ingrained in Data | Enforce Fairness Goals |
| Adebayo, Julius A. “FairML : ToolBox for Diagnosing Bias in Predictive Modeling.” Thesis, Massachusetts Institute of Technology, 2016. https://dspace.mit.edu/handle/1721.1/108212 | Grey Literature (Thesis) | Bias Ingrained in Data | Improve Interpretability or Explainability of Algorithm |
| American Association for the Advancement of Science. Artificial Intelligence and COVID-19: Applications and Impact Assessment. 2021. May 2021. | Grey Literature | Bias Ingrained in Data | Evaluate Disparities in Model Performance |
| D. Valle-Cruz; E. A. Ruvalcaba-Gomez; R. Sandoval-Almazan; J. I. Criado (2019). "A Review of Artificial Intelligence in Government and its Potential from a Public Policy Perspective." 20th Annual International Conference on Digital Government Research (DGO) ():91-99. 10.1145/3325112.3325242 | Grey Literature (Conference Proceedings) | Bias Ingrained in Data | Evaluate Disparities in Model Performance |
| Obermeyer Z, Nissan R, Stern M, Eaneff S, Bembeneck EJ, Mullainathan S. Algorithmic Bias Playbook. The Center for Applied Artificial Intelligence, Chicago Booth;June 2021. | Grey Literature | Bias Ingrained in Data | Evaluate Disparities in Model Performance |
| Osoba, Osonde A., Benjamin Boudreaux, Jessica Saunders, J. Luke Irwin, Pam A. Mueller, and Samantha Cherney, Algorithmic Equity: A Framework for Social Applications. Santa Monica, CA: RAND Corporation, 2019. | Grey Literature | Bias Ingrained in Data | Evaluate Disparities in Model Performance |
| Parikh RB, Teeple S, Navathe AS. Addressing Bias in Artificial Intelligence in Health Care. JAMA. 2019 Dec 24;322(24):2377-2378. doi: 10.1001/jama.2019.18058. PMID: 31755905. | Peer-Reviewed Literature | Bias Ingrained in Data | Evaluate Disparities in Model Performance |
| Rodolfa KT, Salomon E, Haynes L, Mendieta IH, Larson J, Ghani R. Case study: predictive fairness to reduce misdemeanor recidivism through social service interventions. presented at: Proceedings of the 2020 Conference on Fairness, Accountability, and Transparency; 2020; Barcelona, Spain. https://doi.org/10.1145/3351095.3372863 | Grey Literature (Conference Proceedings) | Bias Ingrained in Data | Evaluate Disparities in Model Performance |
| S. H. Park; Y. H. Kim; J. Y. Lee; S. Yoo; C. J. Kim (2019). "Ethical challenges regarding artificial intelligence in medicine from the perspective of scientific editing and peer review." Science Editing 6(2):91-98. 10.6087/kcse.164 | Peer-Reviewed Literature | Bias Ingrained in Data | Evaluate Disparities in Model Performance |
| S. N. Kasturi; J. Park; D. Wild; B. Khan; D. A. Haggstrom; S. Grannis (2021). "Predicting COVID-19-Related Health Care Resource Utilization Across a Statewide Patient Population: Model Development Study." Journal of Medical Internet Research 23(11):10. 10.2196/31337 | Peer-Reviewed Literature | Bias Ingrained in Data | Evaluate Disparities in Model Performance |
| Vokinger KN, Feuerriegel S, Kesselheim AS. Mitigating bias in machine learning for medicine. Communications Medicine. 2021/08/23 2021;1(1):25. doi:10.1038/s43856-021-00028-w | Peer-Reviewed Literature | Bias Ingrained in Data | Evaluate Disparities in Model Performance |
| Z. Obermeyer; B. Powers; C. Vogeli; S. Mullainathan (2019). "Dissecting racial bias in an algorithm used to manage the health of populations." Science 366(6464):447-+. 10.1126/science.aax2342 | Peer-Reviewed Literature | Bias Ingrained in Data | Evaluate Disparities in Model Performance |
| B. G. S. Cruz; M. N. Bossa; J. Solter; A. D. Husch (2021). "Public Covid-19 X-ray datasets and their impact on model bias-A systematic review of a significant problem." Medical Image Analysis 74():16. 10.1016/j.media.2021.102225 | Peer-Reviewed Literature | Bias Ingrained in Data | Increase Model Reporting and Transparency |
| H. Ledford (2019). "Millions of black people affected by racial bias in health-care algorithms." Nature 574, 608-609. 10.1038/d41586-019-03228-6 | Grey Literature (News Media) | Bias Ingrained in Data | Increase Model Reporting and Transparency |
| T. Hernandez-Boussard; S. Bozkurt; J. P. A. Ioannidis; N. H. Shah (2020). "MINIMAR (MINimum Information for Medical Al Reporting): Developing reporting standards for artificial intelligence in health care." Journal of the American Medical Informatics Association 27(12):2011-2015. 10.1093/jamia/ocaa088 | Peer-Reviewed Literature | Bias Ingrained in Data | Increase Model Reporting and Transparency |
| A. Engler (2020). A guide to healthy skepticism of artificial intelligence and coronavirus. Brookings Institution. https://www.brookings.edu/research/a-guide-to-healthy-skepticism-of-artificial-intelligence-and-coronavirus/#cancel | Grey Literature | Bias Ingrained in Data | Avoid or Reduce Use of AI |
| American Association for the Advancement of Science. Artificial Intelligence and COVID-19: Applications and Impact Assessment. 2021. May 2021. | Grey Literature | Bias Ingrained in Data | Improve Diversity, Quality, or Quantity of Data |
| Chen IY, Pierson E, Rose S, Joshi S, Ferryman K, Ghassemi M. Ethical Machine Learning in Healthcare. Annual Review of Biomedical Data Science. 2021;4(1):123-144. doi:10.1146/annurev-biodatasci-092820-114757 | Peer-Reviewed Literature | Bias Ingrained in Data | Improve Diversity, Quality, or Quantity of Data |
| Google. Case Study: Responsible AI: The Fitbit Blood Oxygen Saturation (SpO2) Tracking Feature. https://ai.google/static/documents/case-study-blood-oxygen-saturation-tracking.pdf | Grey Literature | Bias Ingrained in Data | Improve Diversity, Quality, or Quantity of Data |
| Kenneth Holstein, Jennifer Wortman Vaughan, Hal Daumé, Miro Dudik, and Hanna Wallach. 2019. Improving Fairness in Machine Learning Systems: What Do Industry Practitioners Need? In Proceedings of the 2019 CHI Conference on Human Factors in Computing Systems (CHI '19). Association for Computing Machinery, New York, NY, USA, Paper 600, 1–16. DOI:https://doi.org/10.1145/3290605.3300830 | Grey Literature (Conference Proceedings) | Bias Ingrained in Data | Improve Diversity, Quality, or Quantity of Data |
| M. A. Gianfrancesco; S. Tamang; J. Yazdany; G. Schmajuk (2018). "Potential Biases in Machine Learning Algorithms Using Electronic Health Record Data." Jama Internal Medicine 178(11):1544-1547. 10.1001/jamainternmed.2018.3763 | Peer-Reviewed Literature | Bias Ingrained in Data | Improve Diversity, Quality, or Quantity of Data |
| Obermeyer Z, Nissan R, Stern M, Eaneff S, Bembeneck EJ, Mullainathan S. Algorithmic Bias Playbook. The Center for Applied Artificial Intelligence, Chicago Booth;June 2021. | Grey Literature | Bias Ingrained in Data | Improve Diversity, Quality, or Quantity of Data |
| O'Brien N, et al (2022). Addressing racial and ethnic inequities in data-driven health technologies. Imperial College. | Grey Literature | Bias Ingrained in Data | Improve Diversity, Quality, or Quantity of Data |
| Parikh RB, Teeple S, Navathe AS. Addressing Bias in Artificial Intelligence in Health Care. JAMA. 2019 Dec 24;322(24):2377-2378. doi: 10.1001/jama.2019.18058. PMID: 31755905. | Peer-Reviewed Literature | Bias Ingrained in Data | Improve Diversity, Quality, or Quantity of Data |
| Rajkomar A, Hardt M, Howell MD, Corrado G, Chin MH. Ensuring Fairness in Machine Learning to Advance Health Equity. Ann Intern Med. 2018 Dec 18;169(12):866-872. doi: 10.7326/M18-1990. Epub 2018 Dec 4. PMID: 30508424; PMCID: PMC6594166. | Peer-Reviewed Literature | Bias Ingrained in Data | Improve Diversity, Quality, or Quantity of Data |
| Rodolfa KT, Saleiro P, Ghani R. Bias and Fairness (Chapter 11). Big Data and Social Science. 2019. | Grey Literature (Textbook) | Bias Ingrained in Data | Improve Diversity, Quality, or Quantity of Data |
| The Alan Turing Institute. Data science and AI in the age of COVID-19: Reflections on the response of the UK’s data science and AI community to the COVID-19 pandemic. 2021. | Grey Literature | Bias Ingrained in Data | Improve Diversity, Quality, or Quantity of Data |
| V. Goreke; V. Sari; S. Kockanat (2021). "A novel classifier architecture based on deep neural network for COVID-19 detection using laboratory findings." Applied Soft Computing 106():8. 10.1016/j.asoc.2021.107329 | Peer-Reviewed Literature | Bias Ingrained in Data | Improve Diversity, Quality, or Quantity of Data |
| W. Naude; R. Vinuesa (2021). "Data deprivations, data gaps and digital divides: Lessons from the COVID-19 pandemic." Big Data & Society 8(2):12. 10.1177/20539517211025545 | Peer-Reviewed Literature | Bias Ingrained in Data | Improve Diversity, Quality, or Quantity of Data |
| Obermeyer Z, Nissan R, Stern M, Eaneff S, Bembeneck EJ, Mullainathan S. Algorithmic Bias Playbook. The Center for Applied Artificial Intelligence, Chicago Booth;June 2021. | Grey Literature | Bias Ingrained in Data | Use Equity-Focused Checklists, Guidelines, and Similar Tools |
| Delgado C, Baweja M, Crews DC, Eneanya ND, Gadegbeku CA, Inker LA, Mendu ML, Miller WG, Moxey-Mims MM, Roberts GV, St Peter WL, Warfield C, Powe NR. A Unifying Approach for GFR Estimation: Recommendations of the NKF-ASN Task Force on Reassessing the Inclusion of Race in Diagnosing Kidney Disease. J Am Soc Nephrol. 2021 Sep 23;32(12):2994–3015. doi: 10.1681/ASN.2021070988. Epub ahead of print. PMID: 34556489; PMCID: PMC8638402. | Peer-Reviewed Literature | Inclusion of Sensitive Variables | Engage the Broader Community |
| Health Department Launches Coalition to Confront Racism in Medical Algorithms. November 24, 2021, 2021. https://www.nychealthandhospitals.org/pressrelease/health-department-launches-coalition-to-confront-racism-in-medical-algorithms/ | Grey Literature (Press Release) | Inclusion of Sensitive Variables | Improve Governance |
| Darshali A. Vyas, et al. Hidden in Plain Sight — Reconsidering the Use of Race Correction in Clinical Algorithms. N Engl J Med, 0 (June (0)) (2020), 10.1056/NEJMms2004740 | Peer-Reviewed Literature | Inclusion of Sensitive Variables | Exclude Sensitive Variables to Correct for Bias |
| J. Benito-León; M. D. Del Castillo; A. Estirado; R. Ghosh; S. Dubey; J. I. Serrano (2021). "Using Unsupervised Machine Learning to Identify Age- and Sex-Independent Severity Subgroups Among Patients with COVID-19: Observational Longitudinal Study." J Med Internet Res 23(5):e25988. 10.2196/25988 | Peer-Reviewed Literature | Inclusion of Sensitive Variables | Exclude Sensitive Variables to Correct for Bias |
| O'Brien N, et al (2022). Addressing racial and ethnic inequities in data-driven health technologies. Imperial College. | Grey Literature | Inclusion of Sensitive Variables | Exclude Sensitive Variables to Correct for Bias |
| Palmer K. Changing the equation: Researchers remove race from a calculator for childbirth. Stat 2021. | Grey Literature (News Media) | Inclusion of Sensitive Variables | Exclude Sensitive Variables to Correct for Bias |
| Zarsky T. The Trouble with Algorithmic Decisions: An Analytic Road Map to Examine Efficiency and Fairness in Automated and Opaque Decision Making. Science, Technology, & Human Values. 2016;41(1):118-132. doi:10.1177/0162243915605575 | Peer-Reviewed Literature | Inclusion of Sensitive Variables | Exclude Sensitive Variables to Correct for Bias |
| Gaffney T. A yearslong push to remove racist bias from kidney testing gains new ground. *STAT*. 2020-07-17. Accessed December 14, 2022. <https://www.statnews.com/2020/07/17/egfr-race-kidney-test/> | Grey Literature (News Media) | Inclusion of Sensitive Variables | Exclude Sensitive Variables to Correct for Bias |
| #NephJC. Should Race be Replaced? Reconsidering the eGFR Equations. Accessed December 28, 2022, 2022. <http://www.nephjc.com/news/raceandegfr> | Grey Literature | Inclusion of Sensitive Variables | Avoid or Reduce Use of AI |
| Osoba, Osonde A., Benjamin Boudreaux, Jessica Saunders, J. Luke Irwin, Pam A. Mueller, and Samantha Cherney, Algorithmic Equity: A Framework for Social Applications. Santa Monica, CA: RAND Corporation, 2019. | Grey Literature | Exclusion of Sensitive Variables | Improve Governance |
| Schmidt H, Ünver U, Williams M, Pathak P, Sönmez T, Gostin L. Blueprint Labs Discussion Paper #2020.11: What prioritizing worse-off minority groups for COVID-19 vaccines means quantitatively: practical, legal, and ethical implications. 2020. October. | Grey Literature | Exclusion of Sensitive Variables | Improve Governance |
| D. Leslie; A. Mazumder; A. Peppin; M. K. Wolters; A. Hagerty (2021). "Does AI" stand for augmenting inequality in the era of covid-19 healthcare?"." Bmj 372():n304. 10.1136/bmj.n304 | Peer-Reviewed Literature | Exclusion of Sensitive Variables | Include Sensitive Variables to Correct for Bias |
| Veale M, Binns R. Fairer machine learning in the real world: Mitigating discrimination without collecting sensitive data. *Big Data Soc*. 2017/12/01 2017;4(2):2053951717743530. doi:10.1177/2053951717743530 | Peer-Reviewed Literature | Exclusion of Sensitive Variables | Include Sensitive Variables to Correct for Bias |
| Obermeyer Z, Nissan R, Stern M, Eaneff S, Bembeneck EJ, Mullainathan S. Algorithmic Bias Playbook. The Center for Applied Artificial Intelligence, Chicago Booth;June 2021. | Grey Literature | Exclusion of Sensitive Variables | Include Sensitive Variables to Correct for Bias |
| O'Brien N, et al (2022). Addressing racial and ethnic inequities in data-driven health technologies. Imperial College. | Grey Literature | Exclusion of Sensitive Variables | Include Sensitive Variables to Correct for Bias |
| Osoba, Osonde A., Benjamin Boudreaux, Jessica Saunders, J. Luke Irwin, Pam A. Mueller, and Samantha Cherney, Algorithmic Equity: A Framework for Social Applications. Santa Monica, CA: RAND Corporation, 2019. | Grey Literature | Exclusion of Sensitive Variables | Include Sensitive Variables to Correct for Bias |
| Schmidt H, Ünver U, Williams M, Pathak P, Sönmez T, Gostin L. Blueprint Labs Discussion Paper #2020.11: What prioritizing worse-off minority groups for COVID-19 vaccines means quantitatively: practical, legal, and ethical implications. 2020. October. | Grey Literature | Exclusion of Sensitive Variables | Include Sensitive Variables to Correct for Bias |
| Osoba, Osonde A., Benjamin Boudreaux, Jessica Saunders, J. Luke Irwin, Pam A. Mueller, and Samantha Cherney, Algorithmic Equity: A Framework for Social Applications. Santa Monica, CA: RAND Corporation, 2019. | Grey Literature | Exclusion of Sensitive Variables | Evaluate Disparities in Model Performance |
| Rodolfa KT, Saleiro P, Ghani R. Bias and Fairness (Chapter 11). Big Data and Social Science. 2019. | Grey Literature (Textbook) | Exclusion of Sensitive Variables | Evaluate Disparities in Model Performance |
| Margaret Mitchell, et al. Model cards for model reporting. Proceedings of the Conference on Fairness, Accountability, and Transparency, Association for Computing Machinery, ACM Digital Library (2019), pp. 220-229, 10.1145/3287560.3287596 | Grey Literature (Conference Proceedings) | Limited Reporting of Information on Protected Groups | Engage the Broader Community |
| Wu E, Wu K, Daneshjou R, Ouyang D, Ho DE, Zou J. How medical AI devices are evaluated: limitations and recommendations from an analysis of FDA approvals. Nat Med. 2021;27(4):582-584. | Peer-Reviewed Literature | Limited Reporting of Information on Protected Groups | Improve Governance |
| J. Gallifant; J. Zhang; M. Del Pilar Arias Lopez; T. Zhu; L. Camporota; L. A. Celi; F. Formenti (2021). "Artificial intelligence for mechanical ventilation: systematic review of design, reporting standards, and bias." Br J Anaesth ():. 10.1016/j.bja.2021.09.025 | Peer-Reviewed Literature | Limited Reporting of Information on Protected Groups | Evaluate Disparities in Model Performance |
| Wu E, Wu K, Daneshjou R, Ouyang D, Ho DE, Zou J. How medical AI devices are evaluated: limitations and recommendations from an analysis of FDA approvals. Nat Med. 2021;27(4):582-584. | Peer-Reviewed Literature | Limited Reporting of Information on Protected Groups | Evaluate Disparities in Model Performance |
| C. M. Mörch; S. Atsu; W. Cai; X. Li; S. A. Madathil; X. Liu; V. Mai; F. Tamimi; M. A. Dilhac; M. Ducret (2021). "Artificial Intelligence and Ethics in Dentistry: A Scoping Review." J Dent Res 100(13):1452-1460. 10.1177/00220345211013808 | Peer-Reviewed Literature | Limited Reporting of Information on Protected Groups | Increase Model Reporting and Transparency |
| J. Gallifant; J. Zhang; M. Del Pilar Arias Lopez; T. Zhu; L. Camporota; L. A. Celi; F. Formenti (2021). "Artificial intelligence for mechanical ventilation: systematic review of design, reporting standards, and bias." Br J Anaesth ():. 10.1016/j.bja.2021.09.025 | Peer-Reviewed Literature | Limited Reporting of Information on Protected Groups | Increase Model Reporting and Transparency |
| Margaret Mitchell, et al. Model cards for model reporting. Proceedings of the Conference on Fairness, Accountability, and Transparency, Association for Computing Machinery, ACM Digital Library (2019), pp. 220-229, 10.1145/3287560.3287596 | Grey Literature (Conference Proceedings) | Limited Reporting of Information on Protected Groups | Increase Model Reporting and Transparency |
| Wu E, Wu K, Daneshjou R, Ouyang D, Ho DE, Zou J. How medical AI devices are evaluated: limitations and recommendations from an analysis of FDA approvals. Nat Med. 2021;27(4):582-584. | Peer-Reviewed Literature | Limited Reporting of Information on Protected Groups | Increase Model Reporting and Transparency |
| M. Kiener (2020). "Artificial intelligence in medicine and the disclosure of risks." AI Soc ():44205. 10.1007/s00146-020-01085-w | Peer-Reviewed Literature | Algorithms are not interpretable | Train Developers and Users |
| A. M. Antoniadi; Y. H. Du; Y. Guendouz; L. Wei; C. Mazo; B. A. Becker; C. Mooney (2021). "Current Challenges and Future Opportunities for XAI in Machine Learning-Based Clinical Decision Support Systems: A Systematic Review." Applied Sciences-Basel 11(11):23. 10.3390/app11115088 | Peer-Reviewed Literature | Algorithms are not interpretable | Improve Interpretability or Explainability of Algorithm |
| Alejandro Barredo Arrieta, Natalia Díaz-Rodríguez, Javier Del Ser, Adrien Bennetot, Siham Tabik, Alberto Barbado, Salvador Garcia, Sergio Gil-Lopez, Daniel Molina, Richard Benjamins, Raja Chatila, Francisco Herrera. Explainable Artificial Intelligence (XAI): Concepts, taxonomies, opportunities and challenges toward responsible AI. Information Fusion. Volume 58, 2020, Pages 82-115, ISSN 1566-2535, https://doi.org/10.1016/j.inffus.2019.12.012. | Peer-Reviewed Literature | Algorithms are not interpretable | Improve Interpretability or Explainability of Algorithm |
| Osoba, Osonde A., Benjamin Boudreaux, Jessica Saunders, J. Luke Irwin, Pam A. Mueller, and Samantha Cherney, Algorithmic Equity: A Framework for Social Applications. Santa Monica, CA: RAND Corporation, 2019. | Grey Literature | Algorithms are not interpretable | Improve Interpretability or Explainability of Algorithm |
| Reddy; S. Allan; S. Coghlan; P. Cooper (2020). "A governance model for the application of AI in health care." Journal of the American Medical Informatics Association 27(3):491-497. 10.1093/jamia/ocz192 | Peer-Reviewed Literature | Algorithms are not interpretable | Improve Interpretability or Explainability of Algorithm |
| Vokinger KN, Feuerriegel S, Kesselheim AS. Mitigating bias in machine learning for medicine. Communications Medicine. 2021/08/23 2021;1(1):25. doi:10.1038/s43856-021-00028-w | Peer-Reviewed Literature | Algorithms are not interpretable | Improve Interpretability or Explainability of Algorithm |
| Mullainathan S. Biased Algorithms Are Easier to Fix Than Biased People. In: New York Times; 2019. | Grey Literature (News Media) | Algorithms are not interpretable | Evaluate Disparities in Model Performance |
| Cathy O’Neil. Weapons of math destruction: how big data increases inequality and threatens democracy. Crown Publishing Group, USA (2016) | Peer-Reviewed Literature | Algorithms are not interpretable | Avoid or Reduce Use of AI |
| Vokinger KN, Feuerriegel S, Kesselheim AS. Mitigating bias in machine learning for medicine. Communications Medicine. 2021/08/23 2021;1(1):25. doi:10.1038/s43856-021-00028-w | Peer-Reviewed Literature | Algorithms are not interpretable | Avoid or Reduce Use of AI |
| Miller K. When Algorithmic Fairness Fixes Fail: The Case for Keeping Humans in the Loop. Stanford University Human-Centered Artificial Intelligence. https://hai.stanford.edu/news/when-algorithmic-fairness-fixes-fail-case-keeping-humans-loop. Published November 2, 2020. AccessedMarch 8, 2022. | Grey Literature | Optimizing Algorithm Accuracy and Fairness may Conflict | Engage the Broader Community |
| Cathy O’Neil. Weapons of math destruction: how big data increases inequality and threatens democracy. Crown Publishing Group, USA (2016) | Peer-Reviewed Literature | Optimizing Algorithm Accuracy and Fairness may Conflict | Exclude Sensitive Variables to Correct for Bias |
| McCradden MD, Joshi S, Mazwi M, Anderson JA. Ethical limitations of algorithmic fairness solutions in health care machine learning. Lancet Digit Health. 2020;doi:doi:10.1016/S2589-7500(20)30065-0 | Peer-Reviewed Literature | Optimizing Algorithm Accuracy and Fairness may Conflict | Enforce Fairness Goals |
| Rodolfa KT, Lamba H, Ghani R. Empirical observation of negligible fairness–accuracy trade-offs in machine learning for public policy. Nat Mach Intell. 2021/10/01 2021;3(10):896-904. doi:10.1038/s42256-021-00396-x | Peer-Reviewed Literature | Optimizing Algorithm Accuracy and Fairness may Conflict | Enforce Fairness Goals |
| Taylor S., Boniface M., Pickering B., Anderson M., Danks D., Følstad A., Leese M., Müller V., Sorell T., Winfield A., et al. Responsible AI–Key themes, concerns & recommendations for European research and innovation. Zenodo. 2018 doi: 10.5281/zenodo.1303252. | Grey Literature | Optimizing Algorithm Accuracy and Fairness may Conflict | Enforce Fairness Goals |
| A. Chouldechova, A. Roth (2018). The frontiers of fairness in machine learning. Computing research repository (CoRR) Oct. abs/1810.08810. Available at: https://arxiv.org/abs/1810.08810 | Grey Literature (Preprint) | Optimizing Algorithm Accuracy and Fairness may Conflict | Evaluate Disparities in Model Performance |
| McCradden MD, Joshi S, Mazwi M, Anderson JA. Ethical limitations of algorithmic fairness solutions in health care machine learning. Lancet Digit Health. 2020;doi:doi:10.1016/S2589-7500(20)30065-0 | Peer-Reviewed Literature | Optimizing Algorithm Accuracy and Fairness may Conflict | Evaluate Disparities in Model Performance |
| Miller K. When Algorithmic Fairness Fixes Fail: The Case for Keeping Humans in the Loop. Stanford University Human-Centered Artificial Intelligence. https://hai.stanford.edu/news/when-algorithmic-fairness-fixes-fail-case-keeping-humans-loop. Published November 2, 2020. AccessedMarch 8, 2022. | Grey Literature | Optimizing Algorithm Accuracy and Fairness may Conflict | Evaluate Disparities in Model Performance |
| Osoba, Osonde A., Benjamin Boudreaux, Jessica Saunders, J. Luke Irwin, Pam A. Mueller, and Samantha Cherney, Algorithmic Equity: A Framework for Social Applications. Santa Monica, CA: RAND Corporation, 2019. | Grey Literature | Optimizing Algorithm Accuracy and Fairness may Conflict | Evaluate Disparities in Model Performance |
| McCradden MD, Joshi S, Mazwi M, Anderson JA. Ethical limitations of algorithmic fairness solutions in health care machine learning. Lancet Digit Health. 2020;doi:doi:10.1016/S2589-7500(20)30065-0 | Peer-Reviewed Literature | Optimizing Algorithm Accuracy and Fairness may Conflict | Increase Model Reporting and Transparency |
| Osoba, Osonde A., Benjamin Boudreaux, Jessica Saunders, J. Luke Irwin, Pam A. Mueller, and Samantha Cherney, Algorithmic Equity: A Framework for Social Applications. Santa Monica, CA: RAND Corporation, 2019. | Grey Literature | Optimizing Algorithm Accuracy and Fairness may Conflict | Increase Model Reporting and Transparency |
| Miller K. When Algorithmic Fairness Fixes Fail: The Case for Keeping Humans in the Loop. Stanford University Human-Centered Artificial Intelligence. https://hai.stanford.edu/news/when-algorithmic-fairness-fixes-fail-case-keeping-humans-loop. Published November 2, 2020. AccessedMarch 8, 2022. | Grey Literature | Optimizing Algorithm Accuracy and Fairness may Conflict | Avoid or Reduce Use of AI |
| Kenneth Holstein, Jennifer Wortman Vaughan, Hal Daumé, Miro Dudik, and Hanna Wallach. 2019. Improving Fairness in Machine Learning Systems: What Do Industry Practitioners Need? In Proceedings of the 2019 CHI Conference on Human Factors in Computing Systems (CHI '19). Association for Computing Machinery, New York, NY, USA, Paper 600, 1–16. DOI:https://doi.org/10.1145/3290605.3300830 | Grey Literature (Conference Proceedings) | Optimizing Algorithm Accuracy and Fairness may Conflict | Use Equity-Focused Checklists, Guidelines, and Similar Tools |
| Horvitz E, Clyburn M, Griffiths J-M, Matheny J. Privacy and Ethics Recommendations for Computing Applications Developed to Mitigate COVID-19: White Paper Series on Pandemic Response and Preparedness, No. 1. National Center for Advancing Translational Sciences;May 6, 2020. | Grey Literature | Ambiguity in and Conflict among Conceptions of Equity | Engage the Broader Community |
| Osoba, Osonde A., Benjamin Boudreaux, Jessica Saunders, J. Luke Irwin, Pam A. Mueller, and Samantha Cherney, Algorithmic Equity: A Framework for Social Applications. Santa Monica, CA: RAND Corporation, 2019. | Grey Literature | Ambiguity in and Conflict among Conceptions of Equity | Engage the Broader Community |
| Bozkurt S, Cahan EM, Seneviratne MG, Sun R, Lossio-Ventura JA, Ioannidis JPA, Hernandez-Boussard T. Reporting of demographic data and representativeness in machine learning models using electronic health records. J Am Med Inform Assoc. 2020 Dec 9;27(12):1878-1884. doi: 10.1093/jamia/ocaa164. PMID: 32935131; PMCID: PMC7727384. | Peer-Reviewed Literature | Proprietary Algorithms or Data Unavailable for Evaluation | Improve Governance |
| Osoba, Osonde A., Benjamin Boudreaux, Jessica Saunders, J. Luke Irwin, Pam A. Mueller, and Samantha Cherney, Algorithmic Equity: A Framework for Social Applications. Santa Monica, CA: RAND Corporation, 2019. | Grey Literature | Proprietary Algorithms or Data Unavailable for Evaluation | Improve Governance |
| Osoba, Osonde A., Benjamin Boudreaux, Jessica Saunders, J. Luke Irwin, Pam A. Mueller, and Samantha Cherney, Algorithmic Equity: A Framework for Social Applications. Santa Monica, CA: RAND Corporation, 2019. | Grey Literature | Proprietary Algorithms or Data Unavailable for Evaluation | Evaluate Disparities in Model Performance |
| Z. Obermeyer; B. Powers; C. Vogeli; S. Mullainathan (2019). "Dissecting racial bias in an algorithm used to manage the health of populations." Science 366(6464):447-+. 10.1126/science.aax2342 | Peer-Reviewed Literature | Proprietary Algorithms or Data Unavailable for Evaluation | Evaluate Disparities in Model Performance |
| Bowen S, Khoury MJ, Moonesinghe R. “Precision” Health Tools and… Increased Health Disparities? \| Blogs \| CDC. Centers for Disease Control and Prevention, Office of Genomics and Precision Health. https://blogs.cdc.gov/genomics/2020/01/08/precision-health-tools/. Published January 8, 2022. AccessedMarch 8, 2022. | Grey Literature | Proprietary Algorithms or Data Unavailable for Evaluation | Increase Model Reporting and Transparency |
| Margaret Mitchell, et al. Model cards for model reporting. Proceedings of the Conference on Fairness, Accountability, and Transparency, Association for Computing Machinery, ACM Digital Library (2019), pp. 220-229, 10.1145/3287560.3287596 | Grey Literature (Conference Proceedings) | Proprietary Algorithms or Data Unavailable for Evaluation | Increase Model Reporting and Transparency |
| Thinking on its own: AI in the NHS (2018) | Grey Literature | Proprietary Algorithms or Data Unavailable for Evaluation | Increase Model Reporting and Transparency |
| Cathy O’Neil. Weapons of math destruction: how big data increases inequality and threatens democracy. Crown Publishing Group, USA (2016) | Peer-Reviewed Literature | Proprietary Algorithms or Data Unavailable for Evaluation | Avoid or Reduce Use of AI |
| https://www.wired.com/story/health-care-ai-long-way-to-go/ | Grey Literature (News Media) | Proprietary Algorithms or Data Unavailable for Evaluation | Improve Diversity, Quality, or Quantity of Data |
| M. Kiener (2020). "Artificial intelligence in medicine and the disclosure of risks." AI Soc ():44205. 10.1007/s00146-020-01085-w | Peer-Reviewed Literature | Over-Reliance on AI Apps | Train Developers and Users |
| M. A. Gianfrancesco; S. Tamang; J. Yazdany; G. Schmajuk (2018). "Potential Biases in Machine Learning Algorithms Using Electronic Health Record Data." Jama Internal Medicine 178(11):1544-1547. 10.1001/jamainternmed.2018.3763 | Peer-Reviewed Literature | Over-Reliance on AI Apps | Evaluate Disparities in Model Performance |
| A. Engler (2020). A guide to healthy skepticism of artificial intelligence and coronavirus. Brookings Institution. https://www.brookings.edu/research/a-guide-to-healthy-skepticism-of-artificial-intelligence-and-coronavirus/#cancel | Grey Literature | Over-Reliance on AI Apps | Avoid or Reduce Use of AI |
| K. Murphy; E. Di Ruggiero; R. Upshur; D. J. Willison; N. Malhotra; J. C. Cai; N. Malhotra; V. Lui; J. Gibson (2021). "Artificial intelligence for good health: a scoping review of the ethics literature." BMC Med Ethics 22(1):14. 10.1186/s12910-021-00577-8 | Peer-Reviewed Literature | Under-Reliance on AI Apps | Train Developers and Users |
| K. Murphy; E. Di Ruggiero; R. Upshur; D. J. Willison; N. Malhotra; J. C. Cai; N. Malhotra; V. Lui; J. Gibson (2021). "Artificial intelligence for good health: a scoping review of the ethics literature." BMC Med Ethics 22(1):14. 10.1186/s12910-021-00577-8 | Peer-Reviewed Literature | Under-Reliance on AI Apps | Engage the Broader Community |
| K. Ferryman, M. Pitcan (2018). Fairness in Precision Medicine. https://datasociety.net/library/fairness-in-precision-medicine/ | Grey Literature | Repurposing Existing AI Apps Outside Original Scope | Improve Governance |
| Osoba, Osonde A., Benjamin Boudreaux, Jessica Saunders, J. Luke Irwin, Pam A. Mueller, and Samantha Cherney, Algorithmic Equity: A Framework for Social Applications. Santa Monica, CA: RAND Corporation, 2019. | Grey Literature | Repurposing Existing AI Apps Outside Original Scope | Improve Governance |
| D. Leslie; A. Mazumder; A. Peppin; M. K. Wolters; A. Hagerty (2021). "Does AI" stand for augmenting inequality in the era of covid-19 healthcare?"." Bmj 372():n304. 10.1136/bmj.n304 | Peer-Reviewed Literature | Repurposing Existing AI Apps Outside Original Scope | Evaluate Disparities in Model Performance |
| Rodolfa KT, Saleiro P, Ghani R. Bias and Fairness (Chapter 11). Big Data and Social Science. 2019. | Grey Literature (Textbook) | Repurposing Existing AI Apps Outside Original Scope | Evaluate Disparities in Model Performance |
| Margaret Mitchell, et al. Model cards for model reporting. Proceedings of the Conference on Fairness, Accountability, and Transparency, Association for Computing Machinery, ACM Digital Library (2019), pp. 220-229, 10.1145/3287560.3287596 | Grey Literature (Conference Proceedings) | Repurposing Existing AI Apps Outside Original Scope | Increase Model Reporting and Transparency |
| Osoba, Osonde A., Benjamin Boudreaux, Jessica Saunders, J. Luke Irwin, Pam A. Mueller, and Samantha Cherney, Algorithmic Equity: A Framework for Social Applications. Santa Monica, CA: RAND Corporation, 2019. | Grey Literature | Repurposing Existing AI Apps Outside Original Scope | Seek or Provide Restitution for Those Negatively Impacted by AI |
| E. Röösli; B. Rice; T. Hernandez-Boussard (2021). "Bias at warp speed: how AI may contribute to the disparities gap in the time of COVID-19." J Am Med Inform Assoc 28(1):190-192. 10.1093/jamia/ocaa210 | Peer-Reviewed Literature | Application Development or Implementation is Rushed | Increase Model Reporting and Transparency |
| Zimmer M, Franco Z, Madiraju P, Echeveste C, Heindel K, Ogle J. Public Opinion Research on Artificial Intelligence in Public Health Responses: Results of Focus Groups with Four Communities. Washington, DC: AAAS Center for Public Engagement with Science and Technology;2021. | Grey Literature | Unequal Access to AI | Train Developers and Users |
| Aggarwal, N., M. Ahmed, S. Basu, J. J. Curtin, B. J. Evans, M. E. Matheny, S. Nundy, M. P. Sendak, C. Shachar, R. U. Shah, and S. Thadaney-Israni. 2020. Advancing Artificial Intelligence in Health Settings Outside the Hospital and Clinic. NAM Perspectives. Discussion Paper, National Academy of Medicine, Washington, DC. https://doi.org/10.31478/202011f | Grey Literature | Unequal Access to AI | Provide Resources to Those With Less Access to AI |
| C. M. Mörch; S. Atsu; W. Cai; X. Li; S. A. Madathil; X. Liu; V. Mai; F. Tamimi; M. A. Dilhac; M. Ducret (2021). "Artificial Intelligence and Ethics in Dentistry: A Scoping Review." J Dent Res 100(13):1452-1460. 10.1177/00220345211013808 | Peer-Reviewed Literature | Unequal Access to AI | Provide Resources to Those With Less Access to AI |
| IEEE, Ethically Aligned Design: A Vision for Prioriyizing Human Well-being with Autonomous and Intelligence Systems, Version 2.The IEEE Global Initiative on Ethics of Autonomous and Intelligent Systems. Ethically Aligned Design: A Vision for Prioritizing Human Well-being with Autonomous and Intelligent Systems, First Edition. IEEE, 2019. https://standards.ieee.org/content/ieee-standards/en/industry-connections/ec/autonomous-systems.html | Grey Literature | Unequal Access to AI | Provide Resources to Those With Less Access to AI |
| O'Brien N, et al (2022). Addressing racial and ethnic inequities in data-driven health technologies. Imperial College. | Grey Literature | Unequal Access to AI | Provide Resources to Those With Less Access to AI |
| S. Smythe; A. Grotluschen; K. Buddeberg (2021). "The automated literacies of e-recruitment and online services." Studies in the Education of Adults-Niace 53(1):44308. 10.1080/02660830.2020.1855870 | Peer-Reviewed Literature | Unequal Access to AI | Provide Resources to Those With Less Access to AI |
| Whitelaw S, Mamas MA, Topol E, Van Spall HG. Applications of digital technology in COVID-19 pandemic planning and response. The Lancet Digital Health. 2020. | Peer-Reviewed Literature | Unequal Access to AI | Provide Resources to Those With Less Access to AI |
| Aggarwal, N., M. Ahmed, S. Basu, J. J. Curtin, B. J. Evans, M. E. Matheny, S. Nundy, M. P. Sendak, C. Shachar, R. U. Shah, and S. Thadaney-Israni. 2020. Advancing Artificial Intelligence in Health Settings Outside the Hospital and Clinic. NAM Perspectives. Discussion Paper, National Academy of Medicine, Washington, DC. https://doi.org/10.31478/202011f | Grey Literature | Unequal Access to AI | Improve Diversity, Quality, or Quantity of Data |
